# Responsibility to future generations: A strategy for combatting climate change across political divides

**DOI:** 10.1111/bjso.12775

**Published:** 2024-06-20

**Authors:** Stylianos Syropoulos, Kyle Fiore Law, Gordon Kraft‐Todd, Andrea Mah, Ezra Markowitz, Liane Young

**Affiliations:** ^1^ Department of Psychology and Neuroscience Boston College Boston Massachusetts USA; ^2^ The Schiller Institute for Integrated Science and Society Boston College Boston Massachusetts USA; ^3^ Department of Psychology State University of New York, University at Albany Albany New York USA; ^4^ Department of Psychological and Brain Sciences University of Massachusetts Amherst Massachusetts USA; ^5^ Department of Environmental Conservation University of Massachusetts Amherst Massachusetts USA

**Keywords:** climate change, future generations, legacy, moral reframing, responsibility

## Abstract

Individuals and governments often fail to take action to address climate change owing largely to widespread politicization of the issue and related discourse. In response to recent appeals for non‐partisan approaches to pro‐environmentalism, we propose that highlighting one's responsibility to future generations (RFG) could offer promise across the political spectrum. We argue that RFG may be effective because it is widely endorsed, uncorrelated with demographic indicators and less tied to political ideology compared to other forms of responsibility, such as personal responsibility for climate change mitigation. Across six main and seven supplementary studies (*N* = 161,633), we provide evidence for these claims. RFG is not only widely endorsed across countries and demographic groups but it also significantly predicts various measures of pro‐environmental behaviour, both in correlational and pre‐registered experimental contexts. These findings confirm established effects, reconcile inconsistencies and suggest prioritizing intergenerational responsibility may effectively reshape climate change narratives for the most resistant parties.

## BACKGROUND

Anthropogenic climate change increasingly renders the planet inhospitable for life (IPCC, 2022) and poses a long‐term threat to humanity if left unaddressed (MacAskill, 2022). Consequently, the longevity of future generations hinges on people today considering their duty to act on behalf of those to come (Jamieson, 2015). However, even though most people believe in and express concern about (Howe et al., 2015) climate change as an active present‐day threat (van Valkengoed et al., 2023), these concerns often do not translate into tangible action (Gifford, 2011). This is especially so in countries where individual and systemic positions on climate change are polarized along partisan lines, such as the United States (Funk, 2021; Kennedy, 2020; Oreskes & Conway, 2010). The current investigation examines intergenerational concern as a potential mechanism for increasing motivation towards pro‐environmental action that may appeal to individuals across the political spectrum. Building upon emerging findings hinting that concern for the well‐being of future generations may represent a widespread value weakly or not at all related to political identity or partisanship (e.g. Syropoulos & Markowitz, 2021; Zaval et al., 2015) and recent calls to study intergenerational approaches to climate change mitigation (Shrum et al., 2023; Syropoulos & Markowitz, 2023), we examined whether, under what circumstances and for whom emphasizing one's responsibility to future generations may help motivate pro‐environmental engagement.

We put forth two key postulates in reasoning why intergenerational approaches may be effective: relative to perceived personal responsibility to reduce climate change (RCC), feeling a sense of responsibility to future generations (RFG) may be (1) more widely endorsed and (2) less politicized. Nonetheless, intergenerational concerns are likely relatively low salience as people navigate their daily lives and make environmentally relevant decisions, potentially hampering their impact on behaviour in the absence of explicit intervention. Across a total of 13 studies (6 primary studies and 7 supplementary studies; 7 of 13 pre‐registered), we examined support for these postulates. Critically, we also investigated whether RFG predicts greater self‐reported pro‐environmentalism and tested novel and existing interventions targeting responsibility to future generations both directly and by encouraging more abstract reflection in a large‐scale experiment to evaluate the impact of these diverse methods on pro‐environmental outcomes.

### Responsibility to future generations as a pathway to pro‐environmentalism across the political spectrum

Research on intergenerational decision‐making has highlighted the capacity of responsibility‐focused interventions to increase intergenerational beneficence (for review, see Wade‐Benzoni & Tost, 2009; but also see Fox et al., 2010; Wade‐Benzoni, 2019). From such work, there exists suggestive but limited evidence that intergenerational approaches to promoting beneficence could prove particularly successful for increasing climate action and pro‐environmental engagement. Work in this field has primarily focused on the following future‐focused constructs: personal legacy motivations, time orientation and perceived responsibility to future generations (RFG). Extensive work suggests that experimentally manipulating legacy concerns – the degree to which individuals are concerned about being remembered in a positive light following their death – can increase numerous pro‐environmental outcomes (e.g. Grolleau et al., 2021; Hurlstone et al., 2020; Shrum, 2021; Wickersham et al., 2020; Zaval et al., 2015). Similarly, a heightened future time orientation – indicating individuals' inclination to contemplate the future – has consistently demonstrated associations with stronger pro‐environmental attitudes and behaviours across both correlational and experimental studies (e.g. Geiger et al., 2021; Joireman et al., 2004; Park et al., 2020; Soliman et al., 2018).

Yet, while theoretical arguments suggest RFG may help promote pro‐environmental engagement (see Rottman et al., 2015), empirical evidence is scant and tentative. Some correlational research suggests a positive relationship between RFG and pro‐environmental action (Syropoulos et al., 2021; Syropoulos & Markowitz, 2024), but experimental work has been inconclusive (e.g. Watkins & Goodwin, 2020). Thus, emerging inquiry into the effectiveness and robustness across demographic indicators (e.g. political ideology) of diverse approaches to pro‐environmentalism leveraging intergenerational responsibility is still in its infancy. Nevertheless, emerging evidence from a many‐labs investigation testing an intergenerational framing intervention (also evaluated in this study) suggests that these interventions appear relatively effective across a large number of countries (Vlasceanu et al., 2024).

We suggest that intergenerational approaches to pro‐environmentalism may hold promise on account of their potential appeal to a widespread but rarely salient value: an expressed concern to protect future generations. Extant literature hints that RFG may be widely endorsed cross‐nationally at both individual (e.g. Martinez & Winter, 2023) and broader societal levels, with some nations having already enacted policies that explicitly seek to protect the environment on behalf of those to come (e.g. Gonzalez‐Ricoy & Rey, 2019). Moreover, intergenerational responsibility is one of the core tenets of the prominent ethical philosophy and social movement known as longtermism, which positions duty to safeguard the future as society's greatest obligation (MacAskill, 2022; Ord, 2020). While longtermism is a broader philosophical thesis encompassing a greater number of ethical principles than future‐oriented responsibility alone (e.g. longtermism prioritizes utilitarian gains in future welfare over smaller gains for the present), the popularity of longtermist principles among the general populace further suggests potential tractability for intergenerational approaches towards pro‐environmentalism (Law et al., 2023; Syropoulos, Law, Mah, et al., 2024; Syropoulos, Law, & Young, 2024).

Furthermore, we suggest that strategies aimed at fostering RFG may withstand differences in political ideology, as these concerns might not be subject to the same political polarization as responsibility for climate change. We reason this may be so as future generations include the genetic descendants of those living today. Substantial research indicates that individuals, irrespective of their political beliefs, tend to hold genetic relatives in the highest regard in terms of moral standing and obligation (Crimston et al., 2016; McManus et al., 2020), with conservatives showing a particular inclination towards ingroup loyalty (Graham et al., 2017). Evidence and theoretical discussions from the moral reframing literature (e.g. Feinberg & Willer, 2019; Rottman et al., 2015) suggest that non‐partisan support towards politicized issues can be garnered when narratives are framed to align with the moral values of the opposition. We adopt a comprehensive and systematic methodology to evaluate evidence supporting a pathway towards pro‐environmentalism via RFG and the efficacy of varied existing and novel interventions in general and across ideological differences. By doing so, we aim to resolve inconsistencies in the literature and establish a groundwork for future research to explore whether pro‐environmental attitudes and behaviours can be cultivated even among individuals like political conservatives, who typically oppose pro‐environmental initiatives (e.g. by employing moral reframing interventions).

### The current studies

The aims of the current investigation were threefold. First, we sought to investigate the ubiquity of intergenerational responsibility (RFG) across nations and political ideologies. We examined this phenomenon by evaluating how widely prevalent such beliefs are in nationally representative samples across numerous countries in Europe (Study 1), and their *actual* and *perceived* prevalence (descriptive norms) and approval (injunctive norms) across demographic indicators in the United States (Studies 2A‐2B).

Second, we considered whether RFG is more widely endorsed *relative to* perceived responsibility to reduce personal contributions to climate change (RCC; Studies 2A‐2B and 3A‐3B), a core antecedent of pro‐environmental intentions and behaviours evidenced by research (e.g. Helferich et al., 2023; Klockner, 2013; Syropoulos & Markowitz, 2022) informed by norm activation theory (Schwartz, 1977; Schwartz & Howard, 1981) and value–norm–belief theory (Stern, 2000; Stern et al., 1999). Throughout these studies, we also examined associations between RFG and various demographic indicators including political ideology, hypothesizing non‐significant or weak correlations.

Finally, we examined whether feeling more responsible for protecting future generations related to increased self‐reports of pro‐environmentalism (Studies 3A‐3B). We also conducted a large‐scale, highly powered and pre‐registered experiment testing existing and novel interventions targeting RFG (e.g. Shrum, 2021; Watkins & Goodwin, 2020; Zaval et al., 2015) to evaluate the breadth of effective responsibility‐based approaches to increase pro‐environmental engagement and their performance across demographic and ideological divides.

Crucially, these research questions were tested rigorously in a pre‐registered manner, spanning various countries and encompassing diverse sample types such as nationally representative, community‐based, online and student samples, each undergoing several conceptual replications. For all studies, the relevant survey instruments, data files and code are available on the Open Science Framework (OSF), https://osf.io/8s6zw/?view_only=b5af3db79be646cc8fa4b7d7203657dd. All statistical analyses were performed in SAS version 9.4. An overview of all main and supplementary studies is provided in Table 1.

**TABLE 1:** Relevant information for all studies.

Study	Aim	Pre‐registered	Sample type	*M*_age_ (*SD*_age_)	*N*	*N*_Dem. (Rep.)_	*N*_White (POC)_	*N*_female (Male)_	Countries
1	RFG is widely endorsed	Yes	Nationally representative	47.70 (12.01)	109,513	—	—	66,254 (57,248)	34 EU countries
S1A	Conceptual replication of Study 1	No	Nationally representative	42.21 (18.13)	11,729	—	—	6084 (5629)	12 EU countries
S1B	Conceptual replication of Study 1	No	Nationally representative	47.60 (18.19)	30,170	—	—	16,420 (13,750)	31 EU countries
S2	Conceptual replication of Study 1	No	Ecological Society of America	40–49^a^	1215	—	—	396 (817)	USA
2A	RFG is endorsed more than RCC	No	CloudResearch	42.59 (12.79)	457	206 (109)	358 (84)	225 (225)	USA
2B	RFG is endorsed more than RCC	Yes	CloudResearch	42.27 (13.23)	906	430 (197)	692 (165)	438 (452)	USA
S3A	Conceptual replication of Studies 2A‐2B	No	Undergraduates	19.61 (1.74)	628	—	389 (243)	500 (127)	USA
S3B	Conceptual replication of Studies 2A‐2B	No	Undergraduates	19.68 (1.53)	756	—	493 (269)	576 (181)	USA
3A	RFG relates to pro‐environmentalism	Yes	Prolific	35.27 (11.49)	395	209 (50)	274 (121)	193 (193)	USA
3B	RFG relates to pro‐environmentalism	Yes	Prolific	41.69 (14.33)	1800	621 (604)	1436 (364)	865 (886)	USA
S4	Conceptual replication of Studies 3A‐3B	Yes	Community	48.40 (16.89)	328	—	181 (147)	133 (198)	USA
S5	Conceptual replication of Studies 3A‐3B	Yes	Prolific	38.11 (13.74)	561	286 (65)	405 (156)	263 (280)	USA
4	Experimental manipulations increasing RFG	Yes	Prolific	38.50 (13.98)	3175	1654 (469)	2215 (961)	1523 (1569)	USA

Abbreviation: RFG, responsibility to future generations.

^a^ Age was captured in ranges, with the average value falling for the category of ‘40–49’.

## STUDY 1

The goal of our first study was to determine whether there is an overarching concern for future generations. We conducted secondary analyses on data from four rounds of public opinion surveys conducted regularly on behalf of the European Commission and other EU institutions (i.e. Eurobarometers): Eurobarometer 72.4 (October–November 2009), Eurobarometer 73.4 (May 2010), Eurobarometer 72.2 (November–December 2010) and Eurobarometer 75.3 (May 2011). We hypothesized that such concerns would be prevalent in at least half of the population. We also hypothesized that expressing such concerns would not significantly relate to age (Wang et al., 2021), gender (Xiao & McCright, 2013), political ideology (Cruz, 2017) and socioeconomic status (Grandin et al., 2022), all of which have been found to correlate with self‐reports of pro‐environmental attitudes. Analyses and hypotheses for this study were pre‐registered prior to accessing the data, https://aspredicted.org/N4T_GJR.

### Methods

As there were no anticipated temporal differences during the 4 years when the Eurobarometer surveys were conducted (2009–2011), we collapsed across datasets. This decision was facilitated by substantial overlap in countries and measures of interest across these surveys. However, we were unable to utilize the weights provided by the Eurobarometer team due to the merging process, as these weights were unique to each survey wave. Additionally, we pre‐registered our alpha level at <0.001 and deemed correlation coefficients below *r* = .10 as not statistically meaningful, given the substantially large sample sizes. This precaution was taken to prevent practically meaningless coefficients from appearing statistically significant. These decisions were pre‐registered. Given our adjusted alpha, the average sample size for a country in our dataset (*N* = 3966) would provide 99.9% power to detect an effect of *r* = .10 assuming a two‐tailed test.

#### Participants

**FIGURE 1:**
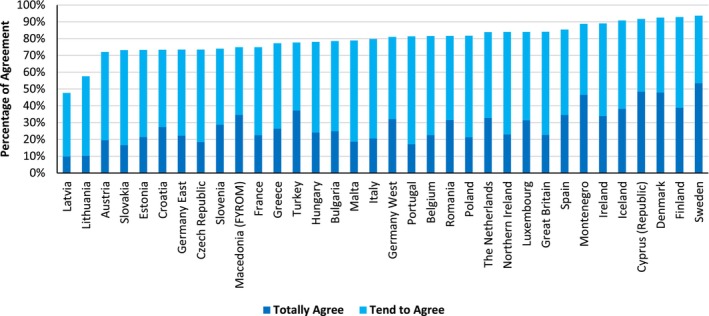
Percentage of participants who tended to or totally agreed with the responsibility to future generations statement.

A total of 109,513 participants were surveyed over the span of 3 years. In total, 34 European countries (see Table S1 in SOM) were included across the four surveys (see Figure 1).

#### Materials

In each Eurobarometer survey, a longer battery of measures was included, pertaining to respondents' views of different social issues. However, only specific variables which were relevant to the present investigation are described below. The full instruments of each Eurobarometer are available online on their respective webpages.

RFG was captured with a single item: ‘We need to reform to benefit future generations even if that means making some sacrifices now’. On a 4‐point Likert scale, 1 for completely disagree and 4 for completely agree. Age was measured in years, and gender was measured in two categories (male and female). Political ideology was measured with a 10‐point Likert scale ranging from 1 = left to 10 = right, and subjective socioeconomic status (SES) was measured with a single item, ‘On the following scale, step “1” corresponds to “the lowest level in the society”; step “10” corresponds to “the highest level in the society.” Could you tell me on which step you would place yourself’?

### Results

Across the 34 countries (and both regions of Germany), reforms to benefit future generations, even at the expense of present generations, were highly supported. We pre‐registered that this level of support would be present if more than 50% of the sample in each country at least tended to agree with the statement. This was the case in 33 of 34 countries, with Latvia being the only exception at 48%. One country was below 60% (Lithuania) and all other countries were above 70%, with a considerable number being above 80% and 90%, respectively (see Figure 1).

#### RFG and demographic indicators

We estimated bivariate (biserial for gender) correlations among age, gender, subjective SES, political ideology and RFG (see Table S2). We then used the methodology suggested by Goh et al. (2016) to estimate (mini) meta‐correlations across all countries. Overall, only the association with SES passed the pre‐registered *r* = .10 threshold to be considered meaningful, as a small positive association was observed (Table 2).

**TABLE 2:** Meta‐correlations of the four demographic indicators and responsibility to future generations.

Variable	Meta‐correlation	Fisher's *Z*	95% CI
Political ideology	*r* = .03	4.35	.017, .045
Socioeconomic status	*r* = .13	18.48	.118, .146
Age	*r* = .07	9.82	.056, .084
Gender (female = 1)	*r* = −.05	−7.56	−.068, −.040

### Discussion

Results from 34 countries across four surveys offer compelling evidence supporting the assertion that people are concerned for the welfare of future generations, even if it means sacrificing benefits for present generations. Furthermore, such concerns appear to have a negligible correlation with political ideology, age and gender. Yet, consistent with research suggesting that poverty‐related stress often places constraints on farsighted decision‐making (Haushofer & Fehr, 2014), a meaningful but weak positive correlation between concern for future generations and SES emerged.

#### Supplementary results – Supplementary Studies S1A‐S1B

We expanded our investigation by examining the prevalence of RFG in additional Eurobarometer surveys. Agreement to a binary measure of RFG varied from 25% to 79% across surveys conducted in 1988 and 2008, encompassing 31 European countries and a total of 41,899 participants (see Studies S1A–S1B in SOM). These surveys focused on participants' willingness to protect the environment for the sake of future generations and utilized different questioning techniques. In one study, interviewers simply noted if participants mentioned future generations, while in the other, participants were presented with a checklist of reasons for addressing climate change, potentially confounding RFG and RCC.

Additionally, we investigated the prevalence of RFG in a sample of members of the American Ecological Society surveyed in 2011 (*N* = 1285). In this sample of highly educated individuals (84% had a PhD/MD), RFG was deemed an important value for a scientist to possess, with 74.1% of scientists considering it a relevant value (see Study S2 in SOM).

## STUDIES 2A‐2B

Having identified evidence for the prevalence of RFG, we investigated individuals' perceptions regarding the extent to which others share this value, as well as their beliefs about whether others consider it morally acceptable to endorse this value. Furthermore, as an initial step in assessing whether an intergenerational approach to pro‐environmentalism could mitigate partisanship on this crucial issue, we explored whether RFG is endorsed to a greater or lesser extent compared to RCC, especially among individuals with a more conservative political ideology. Given that conservatives typically exhibit lower concern for climate change but higher concern for ingroup interests, we hypothesized that this group might demonstrate greater endorsement of RFG compared to RCC. Study 2B was pre‐registered, https://aspredicted.org/T59_81N.

### Methods

#### Participants

##### Study 2A

A total of 457 participants were recruited via CloudResearch (Litman et al., 2017) which operates data collection on MTurk. Participants signed up for a larger study in which they rated public or private acts of generosity, which lasted 10 min and received $1.30 as remuneration.

##### Study 2B

A total of 906 participants were recruited via CloudResearch. Participants signed up for a larger study, which lasted 12 min and received $1.80 as remuneration.

#### Materials and procedure

##### Study 2A

RFG was assessed using a single item (‘When deciding how to live, I have a duty to consider the impact of my actions on future generations’), while RCC was measured with a similar item (‘When deciding how to live, I have a duty to consider the impact of my actions on climate change’). Both items employed the same stem and were rated on a 1–7 scale (1 = strongly disagree, 7 = strongly agree) to ensure that the only distinction was the target of participants' reported sense of responsibility.

Perceived prevalence (descriptive norms) for each type of responsibility – how much participants believed other Americans expressed such perceptions of responsibility – was measured using a single item ranging from 0 to 100 on a slider scale (e.g. ‘What percentage of Americans do you think considers their duty to consider the impact of their actions on future generations when considering how to live their life?’). The presentation order of the two responsibility types was counterbalanced, with half of the participants encountering one type first, providing demographic information and then responding to the questions assessing the other type. Perceived prevalence ratings for each responsibility type were consistently presented after participants expressed their own level of perceived responsibility.

##### Study 2B

Measures relevant to this study were displayed right before the demographic information in a randomized order. RFG and RCC were captured with a single item identical to Study 2A. Perceived prevalence of both responsibility types was measured with two identical items to Study 2A. Perceived moral rightness (injunctive norms) was captured with two items (one per type of responsibility) each using the same stem ‘What percentage of Americans do you think believes that it is morally right to care about the impact of their actions on…[future generations/climate change]’. All items were captured on a slider scale ranging from 0 to 100.

### Results

For all comparisons that follow, we present paired sample *t*‐tests comparing RFG and RCC for each respective category. We also denote bivariate associations (Pearson's correlation) between each instance of RFG and RCC.

#### Comparison of RFG to RCC

##### Study 2A

RCC and RFG were strongly correlated (*r* = .67, *p* < .001). RFG (*M* = 5.48, *SD* = 1.30) was endorsed significantly more (*t*(457) = 5.61, *p* < .001, *d* = 0.26) than RCC (*M* = 5.17, *SD* = 1.58).

##### Study 2B

**FIGURE 2:**
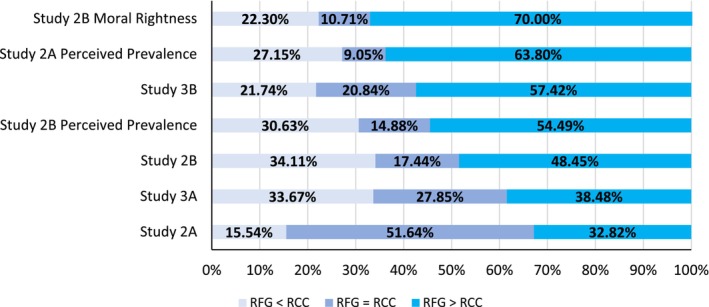
Percentages of participants who personally endorsed and thought that others endorsed responsibility to future generations (RFG) more, less and equal to reduce climate change (RCC). *Note*: For Study 3B, results are reported collapsing across RCC frames. Results are consistent for each individual frame of RCC (see Supplementary Materials.

RCC and RFG were strongly correlated (*r* = .58, *p* < .001). RFG (*M* = 66.51, *SD* = 26.50) was endorsed significantly more (*t*(906) = 7.36, *p* < .001, *d* = 0.26) than RCC (*M* = 60.08, *SD* = 30.54). The distribution of respondents who endorsed one responsibility more, less or equally to the other for all studies is given in Figure 2.

#### Partisan differences in RFG and RCC

##### Study 2A

The difference between RFG and RCC was larger for Republicans than Democrats or Independents. Separate analyses for each group revealed that Republicans expressed greater responsibility towards future generations compared to climate change (*t*(109) = 6.41, *p* < .001, *d* = 0.61), as did Independents, (*t*(132) = 3.71, *p* < .001, *d* = 0.32). However, Democrats did not display such a tendency (*t*(207) = −1.22, *p* = .222).

##### Study 2B

**FIGURE 3:**
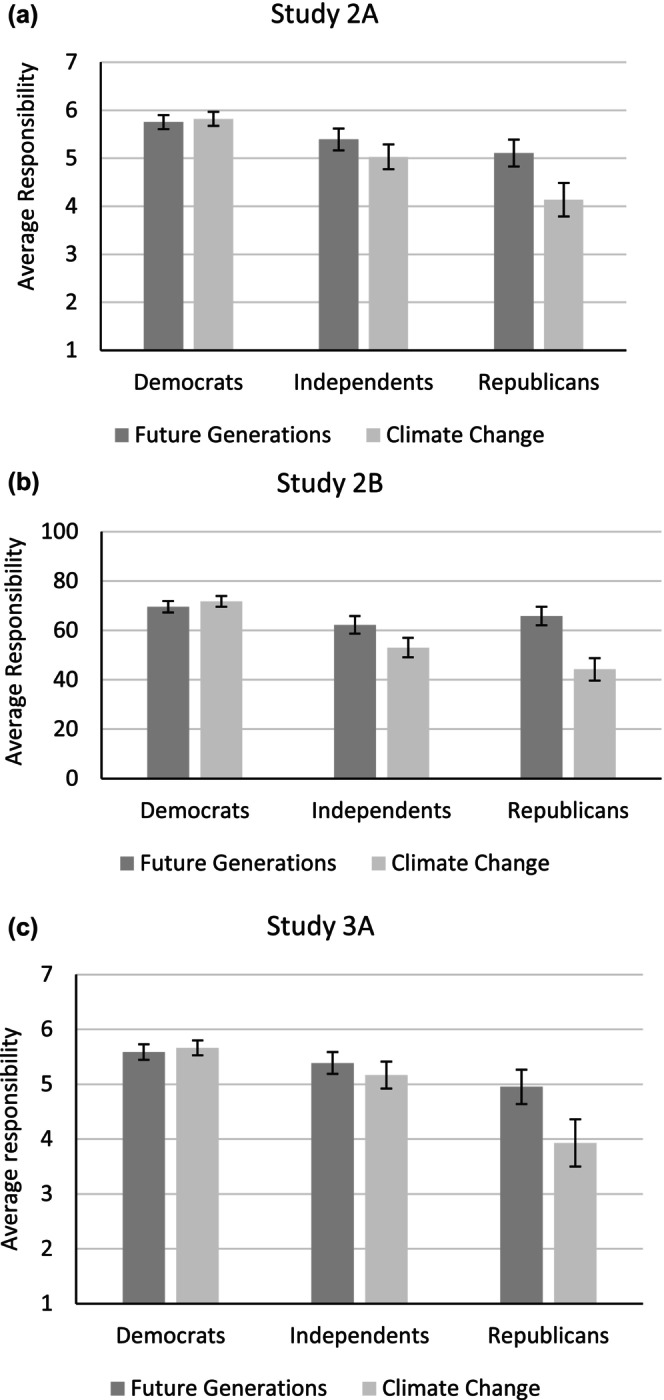
(a‐c) Bar graphs (with 95% CI) depicting differences in perceptions of responsibility for studies 2A‐2B and 3A.

The difference between RFG and RCC was larger for Republicans than Democrats or Independents. Separate analyses for each group revealed that Republicans expressed greater responsibility towards future generations compared to climate change (*t*(196) = 9.31, *p* < .001, *d* = 0.66), as did Independents, (*t*(253) = 5.40, *p* < .001, *d* = 0.34). In Study 2B, Democrats expressed slightly greater responsibility towards climate change (*t*(429) = 2.53, *p* = .012, *d* = 0.12). In both studies, Democrats were the highest on average in both RFG and RCC. These results were also replicated in Study 3A (see SOM; Figure 3).

#### Perceived prevalence of RFG and RCC

##### Study 2A

Similar to personal endorsements of RFG and RCC, perceptions of others' endorsements were strongly correlated (*r* = .69, *p* < .001). Furthermore, participants thought that other Americans expressed greater RFG (*M* = 49.92, *SD* = 21.58) than RCC (*M* = 44.65, *SD* = 20.84, *t*(457) = 6.69, *p* < .001, *d* = 0.31).

##### Studies 2A‐2B

Again, perceptions of others' endorsement were strongly correlated, (*r* = .58, *p* < .001). Furthermore, participants thought that other Americans expressed greater RFG (*M* = 57.48, *SD* = 22.84) than RCC (*M* = 48.59, *SD* = 21.89, *t*(905) = 13.11, *p* < .001, *d* = 0.44).

#### Partisan differences in perceived prevalence of RFG and RCC

##### Study 2A

The aforementioned difference appeared consistently for each political party. Republicans thought that other Americans expressed greater RFG compared to RCC (*t*(109) = 3.80, *p* < .001, *d* = 0.36), as did Independents (*t*(131) = 4.60, *p* < .001, *d* = 0.40) and Democrats (*t*(207) = 3.24, *p* = .001, *d* = 0.22).

##### Study 2B

Again, the aforementioned difference appeared consistently for each political party. Republicans thought that other Americans expressed greater RFG compared to RCC (*t*(196) = 6.63, *p* < .001, *d* = 0.47), as did Independents (*t*(253) = 7.95, *p* < .001, *d* = 0.50) and Democrats (*t*(429) = 8.34, *p* = .001, *d* = 0.40).

#### Differences in perceived moral rightness of RFG and RCC

##### Study 2B

**FIGURE 4:**
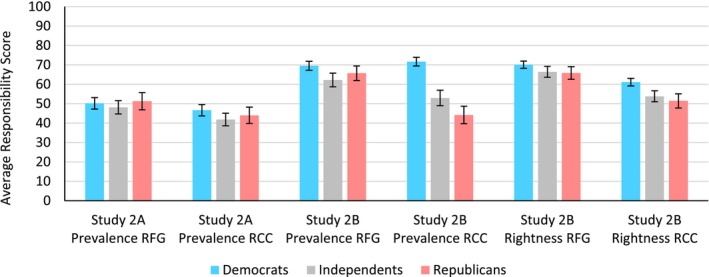
Bar graph (with 95% CI) depicting differences in perceptions of other americans’ endorsement and rightness for each responsibility type for Studies 2A‐2B.

Participants thought that other Americans believed that RFG (*M* = 67.99, *SD* = 21.54) is more morally right compared to RCC (*M* = 56.83, *SD* = 22.70, *t*(905) = 16.64, *p* < .001, *d* = 0.54). Importantly, this difference appears consistently for each political party, such that Republicans thought that other Americans found RFG relative to RCC to be more morally right (*t*(196) = 7.60, *p* < .001, *d* = 0.58), as did Independents, (*t*(253) = 9.23, *p* < .001, *d* = 0.55) and Democrats (*t*(429) = 12.52, *p* = .001, *d* = 0.60, Figure 4).

#### Associations with demographic indicators

Across studies, both types of responsibility were not significantly correlated, or weakly correlated with age, income, education, gender, race and religiosity (see Table 3). Importantly, RFG was weakly correlated with conservative political ideology, while RCC was moderately to strongly and negatively correlated with conservative political ideology. This difference in magnitude between these coefficients was significant and noted in both Study 2A (Fischer's *Z* = 4.00, *p* < .001) and Study 2B (Fischer's *Z* = 7.69, *p* < .001). Meta‐analytical estimates from Studies 2A‐2B and 3A‐3B suggest that RFG correlates with political conservatism at *r* = −.21, while RCC correlates with conservatism at *r* = −.45, suggesting that this association is twice as large. Furthermore, a weak negative correlation between RCC and religiosity was also observed.

**TABLE 3:** Bivariate, biserial and meta‐correlations between RFG and RCC and demographic indicators.

Variable	Study 2A (*N* = 457)		Study 2B (*N* = 906)		Study 3A (*N* = 395)		Study 3B (*N* = 1800)		Meta‐correlations (*N* = 3558)	
	RFG	RCC	RFG	RCC	RFG	RCC	RFG	RCC	RFG	RCC
Age	0.01	0.05	0.12***	−0.04	0.02	−0.05	0.05	0.01	0.06 [0.03, 0.09]	−0.00 [−0.04, 0.03]
Income	0.09	−0.05	0.05	−0.04	0.08	0.02	0.01	−0.03	0.04 [0.01, 0.07]	−0.03 [−0.06, 0.00]
Education	0.10*	0.02	0.02	0.07	0.08	0.12*	0.05	0.04	0.06 [0.03, 0.10]	0.05 [0.02, 0.09]
Gender (Male = 1)	−0.08	−0.09	−0.05	−0.08	−0.04	−0.13**	−0.08	−0.08	−0.07 [−0.10, −0.03]	−0.09 [−.12, −0.05]
Race (White = 1)	−0.03	−0.01	0.01	−0.01	−0.01	0.02	0.01	−0.04	−0.00 [−0.03, 0.03]	−0.02 [−0.05, 0.01]
Conservatism	−0.18***	−0.42***	−0.05	−0.39***	−0.29***	−0.50***	−0.27***	−0.47***	**−0.21 [−0.24, −0.17]**	**−0.45 [−0.48, −0.42]**
Religiosity	0.10***	−0.07	0.11***	−0.08	0.01	−0.14**	−0.00	−0.16***	0.04 [0.01, 0.07]	**−0.13 [−0.16, −0.09]**

Abbreviations: RCC, reduce climate change; RFG, responsibility to future generations.

*Note*: Bolded values highlight significant correlations at *r* > .10. *p < .05, **p < .01, ***p < .001.

### Discussion

Studies 2A‐2B find consistent evidence that people express significantly more RFG compared to RCC. Importantly, such a pattern emerges when we consider the perceived prevalence of each type of responsibility (Studies 2A‐2B), and how morally right each type of responsibility is perceived to be (Study 2B). In addition, consistent with and replicating the results of Study 1 from Europe in the United States, RFG (and RCC) appear to not significantly correlate with most demographic indicators. The only consistent correlation is a weak negative correlation with political conservatism. Importantly, meta‐analytical results including findings from Studies 3A‐3B (total *N* = 3558) suggest that RCC also correlates with conservatism negatively and twice as strongly as RFG and that RCC and not RFG correlated weakly and negatively with religiosity. This evidence aligns well with existing data on perceptions of climate change as a threat, with Republicans (Tyson et al., 2023) and religious Americans (Alper, 2022) tend to be less concerned about the issue compared to their Democrat and secular counterparts. Furthermore, the weaker (for political ideology) and non‐significant (for religiosity) correlation could imply that conservatives and religious Americans could more easily express RFG rather than RCC.

#### Supplementary results – Supplementary Studies S3A‐S3B

We replicated the pattern of results suggesting greater endorsement of RFG than RCC in two separate undergraduate samples (total *N* = 1394) from a large public university. These samples were primarily white, female and climate conscious. However, even in a highly climate‐conscious sample, we found that students expressed more RFG than RCC: (Study S3A: *t*(616) = 7.55, *p* < .001, *d* = 0.30; Study S3B: *t*(751) = 5.71, *p* < .001, *d* = 0.20). See SOM for more information.

## STUDY 3A

Upon discovering that individuals, notably those with conservative views, demonstrate a greater sense of responsibility towards future generations (RFG) than in reducing their own contributions to climate change (RCC), we aimed to explore the relationship between RFG and self‐reported pro‐environmental behaviours. The objective of Study 3A was to assess whether RFG could serve as a viable route to fostering environmentalism, potentially bypassing the partisan divides that often influence perspectives on the issue of climate change in the United States. Moreover, Study 3A served as a pre‐registered replication of Studies 2A‐2B, https://aspredicted.org/58Z_5ND.

### Methods

#### Participants

A total of 395 participants were recruited via Prolific. Participants signed up for a study which lasted 10 min and received $2.00 as remuneration. Two participants were removed due to failing an attention check in accordance with our pre‐registered exclusion criteria.

#### Materials and procedure

Measures were shown to participants in the following groups, with measures within each group presented in a randomized order: (1) responsibility, (2) pro‐environmental outcomes, (3) charity donation and (4) demographic variables. We measured responsibility towards future generations with five items (*a* = .93). The same items were used to measure responsibility to reduce climate change (*a* = .96). See Supplementary Materials for the full set of items.

To measure engagement in pro‐environmental behaviours, we used the self‐report measure created by Brick et al. (2017) (*M* = 2.86, *SD* = 0.51, *a* = .83). This measure captures how frequently participants engage in 21 different pro‐environmental behaviours (e.g. ‘How often do you turn your personal electronics off or in low‐power mode when not in use?’) on a 1–5 Likert scale (1 = never, 5 = always). We also included an item capturing concern about climate change (‘how worried are you about climate change/global warming’; 1 = not at all – 7 = extremely), an item measuring how many years from now participants thought climate change will harm people in the United States, measured on a slider scale ranging from 0 to 100 years in the future, and perceptions of harm from climate change towards: one's self, one's family, people in their community, people in the United States, future generations of people and plants/animal species (1 = not at all to 5 = a great deal). Scores on this measure were averaged across items (*M* = 3.38, *SD* = 0.98, *a* = .94). Finally, we also captured how much people donate to charitable organizations per month in USD.

### Results

#### RFG and pro‐environmental outcomes

RCC and RFG were strongly correlated, *r* = .76, *p* < .001, but RFG (*M* = 5.44, *SD* = 1.10) was endorsed significantly more than RCC (*M* = 5.28, *SD* = 1.34, *t*(394) = 3.65, *p* < .001, *d* = 0.13).^1^ Due to the very high correlation between the two types of responsibility, we ran separate linear regression models for each type of responsibility. Unsurprisingly, participants who felt more RCC also scored higher on pro‐environmental behaviours. For these results, see the Supplementary Materials. We estimated linear regression models with and without demographic covariates. These covariates were as follows: political ideology, education level, income level, age and religiosity. Overall, with and without the addition of demographic covariates, greater RFG predicted increased engagement in pro‐environmental behaviours, concern for climate change, perceptions of harm from climate change and that climate change will harm people in the United States sooner rather than later in the future. Only for existing donations to charity did RFG not have a significant association as the sole predictor. However, with the addition of covariates, some of which significantly correlated with donations, the association between RFG and the outcome became significant.

Findings were similar for RCC, with RCC relating to all outcomes positively, in line with existing research (e.g. Helferich et al., 2023; Klockner, 2013; Syropoulos & Markowitz, 2022). For these results, see the SOM; (Table 4).

**TABLE 4:** Linear regression models with and without covariates for RFG as the predictor.

Outcome	RFG only				RFG and covariates			
	*β*	Lower 95% CI	Upper 95% CI	Adj. *R* ^*2*^	*β*	Lower 95% CI	Upper 95% CI	Adj. *R* ^2^
Pro‐environmental behaviours frequency	.48**	0.19	0.27	.23	.41**	0.15	0.24	.27
Concern for climate change	.52**	0.62	0.86	.27	.37**	0.41	0.64	.44
Years in the future climate change will occur	−.27**	−9.65	−4.52	.07	−.14**	−6.30	−1.26	.22
Climate change will harm people	.50**	0.36	0.51	.25	.36**	0.24	0.38	.39
Donation per month (USD $)	.08	−1.32	11.05	.01	.12*	1.39	14.09	.13

Abbreviation: Adj, adjusted; RFG, responsibility to future generations.

*p < .05, **p < .001.

### Discussion

Study 3A replicated our previous results and expanded on them by providing evidence for a positive association between RFG and self‐reports of pro‐environmentalism. Linear regression models suggested that both types of responsibility appear to contribute to pro‐environmental beliefs and behaviours, although we were not able to test both in the same model due to the high intercorrelations between the two types of responsibility.

#### Supplementary results – Supplementary Study S4

A secondary analysis of data obtained from a field survey in Detroit (*N* = 328), accessed via the Inter‐University Consortium for Political and Social Research (ICPSR 24320 Detroit Area Study, 2002), allowed us to replicate the results of Study 3A, namely evidence that suggests a positive association between RFG and self‐reports of pro‐environmentalism in a community sample. Of a total of 16 pro‐environmental outcomes included in this survey, ranging from pro‐environmental behavioural intentions, environmental concern, climate change risk perception and environmentalist identity (among others), small‐to‐moderate positive associations with RFG were found for 15 outcomes (*β*s ≥ .20, all *p*s ≤ .003, adjusted *R* ^2^ ranged from .05 to .22). All associations remained significant after accounting for age, gender, income, education, conservative political ideology and religiosity. The only outcome for which a non‐significant association was observed was the belief that nature is sacred and should be left alone (see Study S4 in SOM).

## STUDY 3B

Study 3B re‐examined whether RFG relates to self‐reports of pro‐environmentalism. It did so by utilizing a longer battery of measures. Furthermore, we explicitly surveyed a large sample of Democrats, Independents and Republicans in order to observe meaningful associations for each group. Finally, we examined whether people endorse RFG to a greater degree compared to RCC, even when framing RCC in other ways (e.g. addressing global warming, greenhouse gas emissions and carbon emissions). This study was pre‐registered, https://aspredicted.org/BCF_L21.

### Methods

#### Participants

We recruited large sample of 1800 participants via Prolific, to encompass enough participants for the comparison of responsibility to future generations and responsibility to reduce climate change, phrased in four different ways (i.e. climate change, carbon emissions, greenhouse gas emissions and global warming), and to be adequately powered for separate analyses by political group (Republican, Democrat and Independents). We screened participants to recruit 600 Republicans, 600 Democrats and 600 Independents based on Prolific's screening questions. We did this purposely so that we could meaningfully examine associations for each political group. Participants signed up for a study which lasted 4 min and received $0.80 as remuneration. Seven participants were removed due to failing an attention check.

#### Materials and procedure

Participants were randomly assigned to one of four conditions. Regardless of the condition they were assigned to, they were first presented with the measures of responsibility (to future generations and to reducing climate change/carbon emissions/greenhouse gas emissions/global warming). Importantly, both of these measures were displayed on the same page and on the same scale (ranging from 0 = strongly disagree to 100 = strongly agree). Then, they completed a short, three‐item version of the following subscales of the Environmental Attitudes Inventory (Milfont & Duckitt, 2010): interventionist conservation policies (*M* = 5.00, *SD* = 1.49, *a* = .86), environmental concern (*M* = 5.93, *SD* = 1.00, *a* = .71), environmental threat (*M* = 5.46, *SD* = 1.38, *a* = .86), personal conservation behaviours (*M* = 5.53, *SD* = 1.19, *a* = .88) and environmental movement activism (*M* = 4.05, *SD* = 1.64, *a* = .88). They then provided some basic demographic information and were subsequently debriefed.

### Results

#### Comparison of RFG to RCC

Responsibility to reduce climate change, regardless of how it was framed, and responsibility towards future generations were strongly correlated, with *r*s ranging from *r* = .67 to *r* = .75, all *p*s < .001. Across all participants, regardless of how responsibility to reduce climate change was framed, participants reported significantly more responsibility to protect future generations (see Table 5).^2^ Thus, results from our previous studies were replicated across all possible frames of climate change.

**TABLE 5:** Comparisons of the two types of responsibility across the different frames for all groups.

Comparison of RFG with…	Full sample		Democrats		Independents		Republicans	
	*t*‐test (df)	*d*	*t*‐test (df)	*d*	*t*‐test (df)	*d*	*t*‐test (df)	*d*
… responsibility for reducing Global Warming	*t*(437) = 7.73***	0.37	*t*(151) = 0.99	0.08	*t*(130) = 3.71***	0.32	*t*(148) = 7.28***	0.60
… responsibility for reducing Greenhouse Gas Emissions	*t*(448) = 8.83***	0.42	*t*(159) = 0.95	0.07	*t*(137) = 4.54***	0.39	*t*(150) = 9.47***	0.77
… responsibility for reducing Carbon Emissions	*t*(444) = 11.96***	0.56	*t*(1543 = 4.95***	0.40	*t*(136) = 5.25***	0.45	*t*(149) = 10.17***	0.83
… responsibility for reducing Climate Change	*t*(447) = 9.03***	0.43	*t*(154) = 2.83**	0.23	*t*(137) = 4.74***	0.40	*t*(152) = 7.61***	0.61

**p < .01, ***p < .001.

#### Partisan differences in RFG and RCC

**FIGURE 5:**
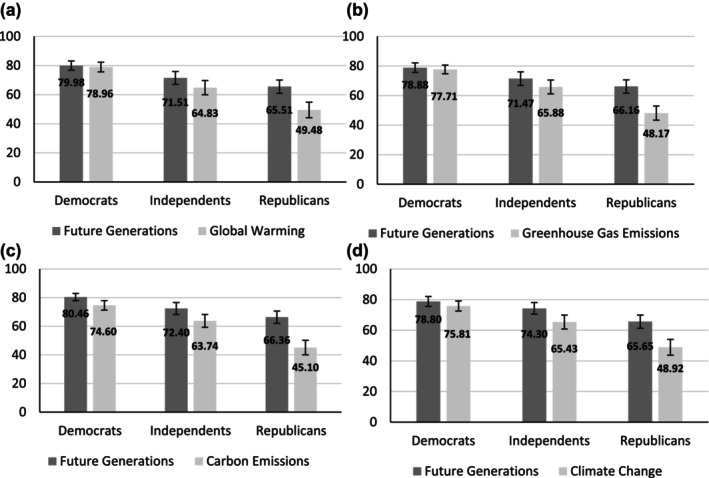
(a‐d) Bar graphs with 95% CI for comparisons of the two types of responsibility across all four frames for republicans, democrats and independents. (a) presents these comparisons with respect to the “Global Warming” frame, (b) with respect to the “Greenhouse Gas Emissions” frame, (c) with respect to the “Carbon Emissions” frame, and (d) with respect to the “Climate Change” frame.

Replicating the findings from our previous studies, Republicans and Independents reported greater RFG compared to RCC for each of the four frames. Democrats also reported greater RFG compared to RCC for two of four frames (carbon emissions and climate change). Across the board, Democrats scored higher than Independents and Republicans in RFG and RCC (Figure 5).

#### RFG and pro‐environmental outcomes

Again, due to the very high correlation between the two types of responsibility, we ran separate linear regression models for each type of responsibility. Similar to Study 3A, those who felt more RCC also scored higher on all pro‐environmental outcomes. For these results, see Table S19 in the SOM. We estimated linear regression models without and with demographic covariates. These covariates were as follows: political ideology, education level, income level, age and religiosity.^3^

Overall, with and without the addition of demographic covariates, greater RFG predicted increased support for interventionist conservation policies, environmental concern, environmental threat, personal conservation behaviours and environmental movement activism (Table 6).

**TABLE 6:** Linear regression models with and without covariates for responsibility to future generations as a predictor.

Outcome	RFG only				RFG and covariates			
	*β*	Lower 95% CI	Upper 95% CI	Adj. *R* ^2^	*β*	Lower 95% CI	Upper 95% CI	Adj. *R* ^2^
Support for interventionist conservation policies	.47*	0.026	0.031	.22	.35*	0.019	0.023	.44
Environmental concern	.48*	0.018	0.021	.23	.40*	0.015	0.018	.32
Environmental threat	.49*	0.025	0.030	.24	.38*	0.019	0.023	.43
Personal conservation behaviours	.54*	0.024	0.028	.29	.49*	0.022	0.025	.33
Environmental movement activism	.54*	0.033	0.038	.29	.46*	0.028	0.033	.39

Abbreviation: Adj, adjusted.

* *p* < .001.

### Discussion

Regardless of how we framed different aspects of climate change, our results suggest that, on average, Americans report feeling more RFG than RCC. This effect held when both types of responsibility were shown simultaneously to participants and were primarily driven by Independents and Republicans who felt more responsible for protecting future generations than they did for addressing climate change. Crucially, when we look at overall endorsement of RFG and RCC, Democrats scored the highest and did not differ in endorsement of these two types of responsibility.

Furthermore, for each of the three political groups, feeling more responsible for future generations related to each of five pro‐environmental outcomes included in the study, explaining between 22% and 29% of the variance and remaining a significant predictor even after accounting for different demographic indicators. Having amassed extensive evidence for the prevalence of people's responsibility to future generations,^4^ its relatively depoliticized nature and its association with a host of pro‐environmental outcomes, in our final study, we sought to experimentally manipulate this mechanism.

#### Supplementary results – Supplementary Study 5

An additional study (Study S5 in SOM) was also conducted on Prolific. This study aimed to test the same hypothesis as Study 3B. However, due to an unexpected but significant order effect in our survey, and a very low number of Republicans in our sample (65 of 561 participants), we were unable to compare the different types of responsibility. Again, RFG was associated with greater scores for personal conservation behaviours (*β* = .40, *p* < .001), support for conservation policies (*β* = .58, *p* < .001), environmental concern (*β* = .45, *p* < .001), perceived threat to the environment (*β* = .53, *p* < .001) and lower scores for protecting the environment for anthropocentric reasons (*β* = −.35, *p* < .001), even after adjusting for demographic covariates.

## STUDY 4

Our final study aimed to experimentally manipulate RFG. Previous research has endeavoured to manipulate RFG (e.g. Watkins & Goodwin, 2020) or related constructs such as legacy motivations (e.g. Shrum, 2021; Zaval et al., 2015). Building upon this earlier work, we aimed to conduct both a pre‐registered (conceptual) replication of existing research and to explore novel approaches to enhancing people's sense of responsibility to future generations. Crucially, Study 4 aimed to explore a causal relationship between RFG and pro‐environmental outcomes. It also aimed to assess the effectiveness of various interventions to determine whether RFG and its influence on pro‐environmental behaviour are adaptable and can be targeted in diverse ways and across ideological divides. This study was pre‐registered, https://aspredicted.org/14N_T2T.

### Methods

#### Participants

This study tested five different manipulations encouraging both more direct (e.g. reading and writing tasks) as well as more abstract and subtle (e.g. merely watching a brief video) reflections. Our focus was on comparing each of these manipulations relative to a control condition. Thus, rather than conducting an a priori power analysis for an omnibus effect, we estimated a power analysis for a *t*‐test with two independent means, a small effect size (*d* = 0.20) and power set to 0.90, assuming equal sample sizes for each condition. We set our sample size to *d* = 0.20 because previous research found effect sizes around this magnitude, and due to established norms about small and meaningful effect sizes. A sample of 527 participants per condition was large enough to detect effect sizes of *d* = 0.20. We rounded this number up to 540 participants per condition. Multiplying this number by the number of conditions (i.e. a total of six) we reached a sample of 3240 participants across all conditions. We collected data via Prolific. Participants signed up for a study, which lasted 7 min and received $1.20 as remuneration. Per our pre‐registered exclusion criteria, 67 participants were removed, leaving a total of 3175 participants.

#### Procedure

Participants were randomly assigned to one of six conditions described below. Materials for each condition are available on OSF.

##### Letter

In the letter condition (*N* = 478), participants completed a modified version of the manipulation created by Shrum (2021). In this condition, participants are asked to write a letter to a person born today (i.e. 2022), who will be 28 years old in the year 2050. In this letter, participants are telling this person what they (i.e. the participant) can do today to help create a sustainable future for them (i.e. the person who will be 28 years old in 2050). We opted to use this adapted version of the manipulation, as it allows us to use this intervention regardless of one's parental status while having the same target recipient in mind. A similar version of this intervention was tested by Vlasceanu et al. (2024).

##### Legacy

This condition (*N* = 527) was identical to the legacy reflection exercise used by Zaval et al. (2015). Participants were asked to reflect on and write about the legacy they want to leave behind, as well as the skills and traits they would want to pass onto others.

##### Sacrifice

Participants in the sacrifice condition (*N* = 514) were shown instructions that were identical to those of Watkins and Goodwin (2020, Study 1). In particular, participants were asked to reflect on and write about the sacrifices made by members of the past generation and how they (i.e. the participant) benefited from them.

##### Video

Participants in the video condition (*N* = 553) saw a 1‐min video about how we should try to preserve a national park (in this case, the Grand Canyon) for future generations. This video is available online and was produced by the National Parks Foundation: https://www.grandcanyontrust.org/keep‐the‐canyon‐grand.

##### Longtermism

Participants in the longtermism condition (*N* = 555) read an excerpt from the book ‘What We Owe The Future’ (MacAskill, 2022). This section was 385 words, and was taken from the introduction of the book at the beginning of the section ‘Future People Count’.

##### Control

Participants in the control condition (*N* = 552) were instructed to write about their daily routine in the morning after they wake up and in the evening hours before they went to bed.

Regardless of condition, after completing the relevant condition‐specific task, participants responded to the different outcomes (RFG, RCC, intentions, policy support and donation task) in a randomized order.

#### Materials

##### Responsibility

Three items (*a* = .92) on a 7‐point Likert scale (1 = strongly disagree, 7 = strongly agree) were used to capture perceived responsibility to future generations. These items, on the same 7‐point Likert scale, were slightly modified to capture responsibility for reducing climate change (*a* = .94).

##### Pro‐environmental intentions

Six items (*a* = .77), on 6‐point Likert‐type scale (1 = never, 6 = all the time) taken directly from Zaval et al. (2015) were used to capture self‐reports of pro‐environmental intentions for the next 3 months.

##### Pro‐environmental policy support

Six items (*a* = .85), on 6‐point Likert scale (1 = strongly oppose, 6 = strongly support) taken directly from Zaval et al. (2015), were used to capture pro‐environmental intentions for the next 3 months.

##### Donation task

Finally, participants were also instructed that one participant would receive a $10 bonus as a thank you for participating in the study. Participants were given the option to keep this $10 prize or to donate part or all of it to a charity. We chose Trees for the Future as the designated charity for this task. Participants were given a brief description of Trees for the Future and were asked to allocate any amount from the $10 to themselves or the charity. This measure was taken directly from Zaval et al. (2015).

### Results

**FIGURES 6:**
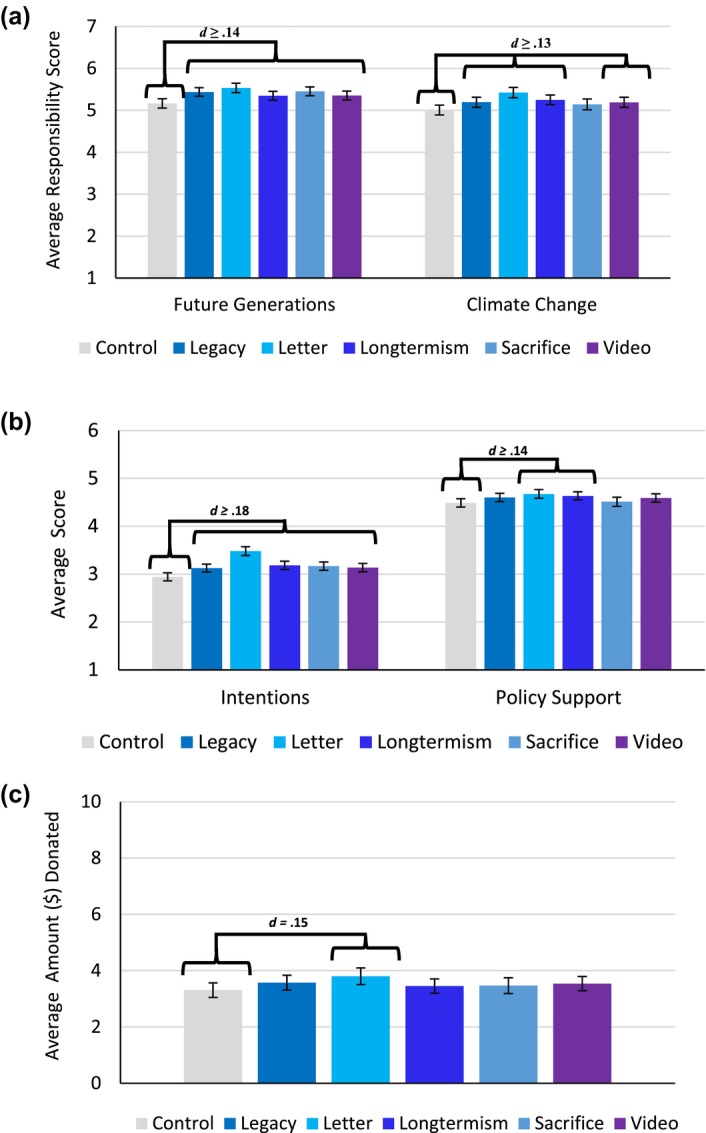
Bar graphs depicting scores for all outcomes with 95% CI for each condition. (a) depicts condition differences for responsibility, (b) for intentions and policy support, and (c) for amount donated.

We pre‐registered analyses comparing each condition to the control. We chose this strategy, as our hypothesis was that the five experimental conditions would increase each of the five outcomes relative to the control. To determine this difference, we ran a series of five linear regression models, one per outcome of interest. In each regression model, five dummy‐coded variables representing each experimental condition were entered as simultaneous predictors. Thus, each dummy‐coded predictor showed the difference in that specific condition relative to the control. Finally, in each model, the intercept indicates the value for the control. These results are summarized in Table 7 and depicted visually in Figure 6a–c. Importantly, results are robust to the inclusion of demographic covariates (see Table S21 in SOM; for correlations between demographic variables and all measures, see Table S20 in SOM).

**TABLE 7:** Results for the effect of each condition on all outcomes.

	*b*	*β*	*SE*	*t*	*p*	*d*	95% CI	
Donations to charity								
Intercept (control)	3.31	.00	0.13	24.89	<.001		3.05	3.57
Legacy	0.26	.03	0.19	1.39	.165	0.086	−0.11	0.64
Letter	**0.49**	**.06**	**0.20**	**2.51**	**.012**	**0.154**	**0.11**	**0.87**
Longtermism	0.14	.02	0.19	0.77	.443	0.047	−0.22	0.51
Sacrifice	0.16	.02	0.19	0.85	.397	0.051	−0.21	0.54
Video	0.23	.03	0.19	1.23	.220	0.075	−0.14	0.60
Policy support								
Intercept (control)	4.49	.00	0.04	101.37	<.001		4.40	4.57
Legacy	0.11	.04	0.06	1.79	.073	0.109	−0.01	0.24
Letter	**0.19**	**.06**	**0.07**	**2.87**	**.004**	**0.183**	**0.06**	**0.31**
Longtermism	**0.15**	**.05**	**0.06**	**2.35**	**.019**	**0.142**	**0.02**	**0.27**
Sacrifice	0.03	.01	0.06	0.41	.684	0.024	−0.10	0.15
Video	0.10	.04	0.06	1.66	.097	0.100	−0.02	0.23
Pro‐environmental intentions								
Intercept (control)	2.94	.00	0.04	67.89	<.001		2.86	3.03
Legacy	**0.18**	**.06**	**0.06**	**2.92**	**.004**	**0.184**	**0.06**	**0.30**
Letter	**0.54**	**\.19**	**0.06**	**8.46**	**<.001**	**0.539**	**0.41**	**0.66**
Longtermism	**0.24**	**.09**	**0.06**	**3.92**	**<.001**	**0.236**	**0.12**	**0.36**
Sacrifice	**0.22**	**.08**	**0.06**	**3.58**	**<.001**	**0.221**	**0.10**	**0.35**
Video	**0.19**	**.07**	**0.06**	**3.15**	**.002**	**0.187**	**0.07**	**0.31**
Responsibility to reduce climate change								
Intercept (control)	5.01	.00	0.06	82.43	<.001		4.89	5.13
Legacy	**0.19**	**.05**	**0.09**	**2.14**	**.033**	**0.130**	**0.02**	**0.36**
Letter	**0.42**	**.10**	**0.09**	**4.67**	**<.001**	**0.299**	**0.24**	**0.59**
Longtermism	**0.24**	**.06**	**0.09**	**2.84**	**.005**	**0.174**	**0.08**	**0.41**
Sacrifice	0.14	.03	0.09	1.57	.118	0.094	−0.03	0.31
Video	**0.19**	**.05**	**0.09**	**2.15**	**.031**	**0.129**	**0.02**	**0.35**
Responsibility to future generations								
Intercept (control)	5.17	.00	0.05	96.24	<.001		5.06	5.27
Legacy	**0.27**	**.08**	**0.08**	**3.54**	**<.001**	**0.213**	**0.12**	**0.42**
Letter	**0.37**	**.10**	**0.08**	**4.70**	**<.001**	**0.288**	**0.22**	**0.53**
Longtermism	**0.18**	**.05**	**0.08**	**2.38**	**.017**	**0.140**	**0.03**	**0.33**
Sacrifice	**0.29**	**.08**	**0.08**	**3.73**	**<.001**	**0.224**	**0.14**	**0.44**
Video	**0.19**	**.06**	**0.08**	**2.51**	**.012**	**0.147**	**0.04**	**0.34**

*Note*: Bolded values highlight significant results.

**FIGURE 7:**
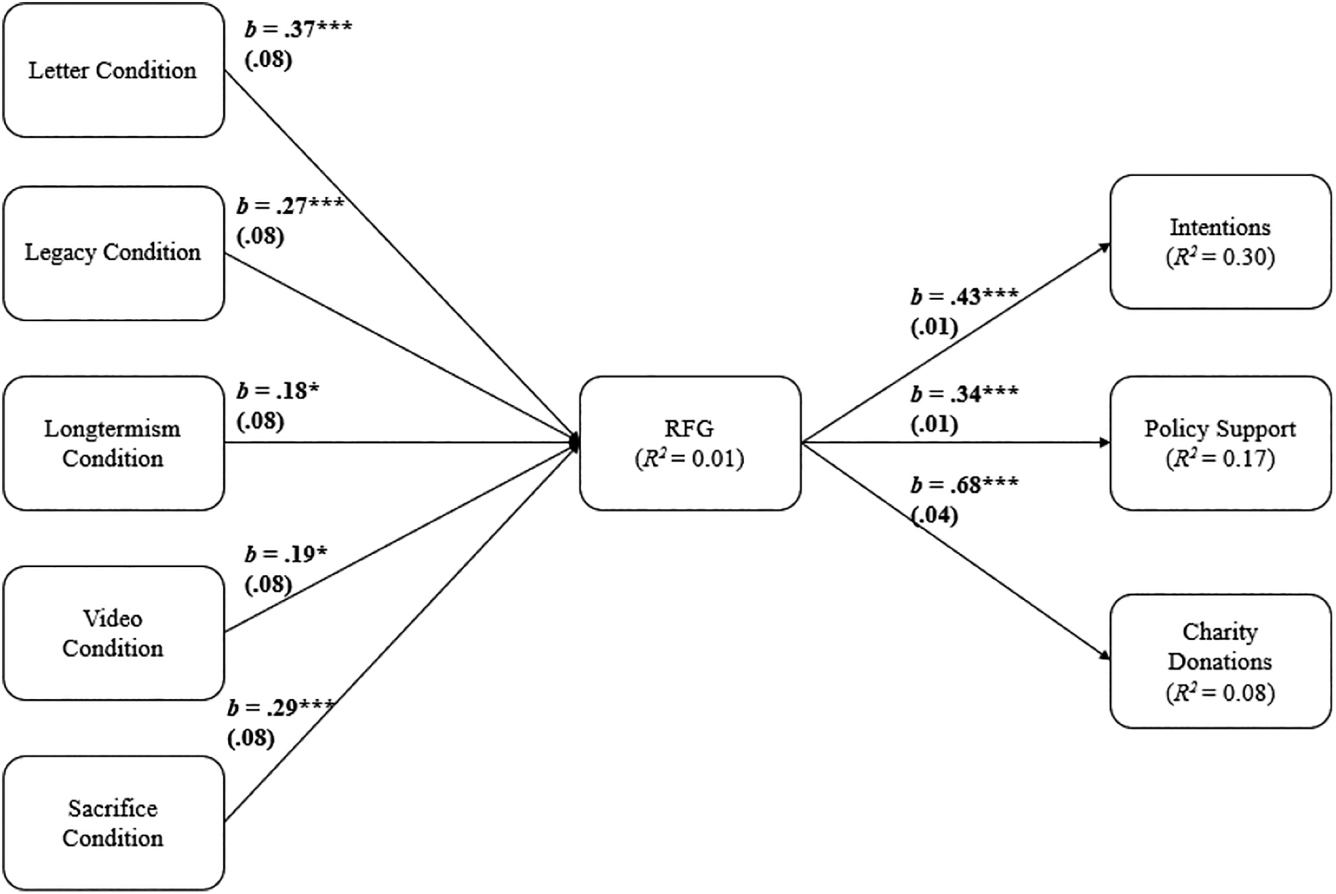
Pre‐registered path model with RFG as a mediator. *Note*: Unstandardized weights are displayed. *p < .05, ***p < .001.

#### Responsibility to future generations

Supporting our hypothesis, all conditions significantly increased RFG relative to the control condition. Importantly, this test served as a replication of Zaval et al. (2015), Shrum (2021) and Watkins and Goodwin (2020).

#### Responsibility to reduce climate change

All conditions except for the sacrifice condition significantly increased RCC. Importantly, this test served as a conceptual replication of Zaval et al. (2015). This measure was not included in Watkins and Goodwin (2020). Given that this manipulation did not focus on climate change, or the environment, but rather focused on the sacrifices made by previous generations more broadly, it is perhaps not surprising that participants in this condition only reported feeling more responsible towards future generations and did not feel more responsible for reducing climate change. Furthermore, the fact that we were able to shift responsibility to future generations without shifting responsibility to reduce climate change adds to our argument that these are two separate mechanisms, despite their high correlation.

#### Pro‐environmental intentions

Supporting our hypothesis, all conditions significantly increased self‐reports of pro‐environmental intentions for the next 3 months. Importantly, this test served as a replication of Zaval et al. (2015) and Shrum (2021). A measure of pro‐environmental intentions was not included in Watkins and Goodwin (2020).

#### Pro‐environmental policy support

Only the letter and longtermism conditions significantly increased support for pro‐environmental policies. Effects for the legacy condition were marginal, failing to replicate the findings of Zaval et al. (2015). Similar to Watkins and Goodwin, we did not find a significant effect of sacrifice manipulation on policy support.

#### Donations to charity

Only the letter condition significantly increased donations to charity, thus replicating the results of Shrum (2021), and failing to replicate Zaval et al. (2015).

#### Robustness of results

Per our pre‐registered analytical plan, we also ran analyses controlling for demographic variables that were significantly related to our outcomes or variables for which there was an error of random assignment. There were no differences in any demographic variables across conditions, suggesting that there was no error in random assignment. Furthermore, when including any demographic variables that related to outcomes at *r* > .10, results remain highly consistent and significant (see Supplementary Materials for further information).

#### Exploratory analysis: Indirect effects via responsibility to future generations

We pre‐registered an exploratory analysis in which responsibility to future generations would act as a mediator for the effect of condition on all outcomes. This analysis was conducted in all existing investigations: Zaval et al. (2015) and Shrum (2021) treated legacy concerns (i.e. whether one is seen by future generations positively) as a mediator and Watkins and Goodwin (2020) treated obligation to future generations as a mediator. Mirroring this approach, we estimated a path model with the five dummy‐coded variables for each condition (i.e. condition = 1, control = 0) as exogenous variables, RFG as a mediator^5^ and the three outcomes (intentions, policy support and donations) as parallel outcomes. This model is akin to a mediation model and thus it was fully saturated (i.e. it had zero degrees of freedom, and thus produced no fit indexes). However, in the same model, if the non‐significant effects of condition on the outcomes are trimmed from the model, the resulting model fit meets the criteria (e.g. Kline, 2015) for good model fit: *χ* ^*2*^(7) = 0.69, *p* = .686, CFI = 0.999, RMSEA < 0.001, SRMR = 0.006 (Table 8).

**TABLE 8:** Indirect effects via increased RFG of condition relative to control tested in Figure 7.

Predictor (condition)	Outcome	*b*	*β*	Lower 95% CI	Upper 95% CI	*p*
Video	Donation	0.13	.02	0.03	0.23	.013
Sacrifice	Donation	0.20	.02	0.09	0.30	<.001
Longtermism	Donation	0.12	.02	0.02	0.22	.018
Letter	Donation	0.25	.03	0.14	0.36	<.001
Legacy	Donation	0.18	.02	0.29	0.14	<.001
Video	Intentions	0.08	.03	0.02	0.14	.012
Sacrifice	Intentions	0.12	.04	0.06	0.19	<.001
Longtermism	Intentions	0.08	.03	0.01	0.14	.017
Letter	Intentions	0.16	.05	0.09	0.22	<.001
Legacy	Intentions	0.12	.04	0.05	0.18	<.001
Video	Policy Support	0.06	.02	0.01	0.11	.012
Sacrifice	Policy Support	0.10	.03	0.05	0.15	<.001
Longtermism	Policy Support	0.06	.02	0.01	0.11	.018
Letter	Policy Support	0.13	.04	0.07	0.17	<.001
Legacy	Policy Support	0.09	.03	0.04	0.14	<.001

Importantly, in this model, the effects of each condition (relative to the control) on the mediator were significant, such that each condition significantly increased responsibility to future generations. In turn, RFG was also significantly and positively related to each of the three outcomes. Importantly, all indirect effects of condition on each of the three outcomes, via increased RFG, were significant and positive.

### Discussion

Our final study tested the effectiveness of five different interventions, each aiming to increase responsibility to future generations. Three of these interventions have been validated in past work (sacrifice: Watkins & Goodwin, 2020; letter: Shrum, 2021; legacy: Zaval et al., 2015), while two were created for this study (longtermism and video) with an eye towards applicability and scalability. Our results suggest that each of these interventions can indeed increase RFG and pro‐environmental intentions. Furthermore, each intervention except for the reflection on sacrifices made by past generations also increased RCC, even though two of the interventions did not mention climate change (i.e. legacy and longtermism). Moreover, only the letter, legacy and longtermism interventions successfully increased support for policy, with the letter also being the only intervention that increased donations to a charity. Considering these results, it appears that the letter intervention is the most consistent method of both increasing responsibility to future people and pro‐environmental intentions and behaviours (in the lab), a finding that mirrors results from cross‐national investigations well (see Vlasceanu et al., 2024). Importantly, all conditions (relative to the control) had significant indirect effects on all three outcomes, through increased responsibility to future generations.

Although the effects noted were mostly small, these results partially replicate existing investigations and also highlight that interventions focusing on engendering the longtermism philosophy could prove successful in shifting intergenerational concern (i.e. RFG). Furthermore, the fact that a 1‐min video was also effective proves promising for developing interventions that do not rely on reflections (such as the letter, legacy and sacrifice interventions). Ultimately, these results suggest that it is possible to enhance people's sense of responsibility to future generations through diverse approaches and that doing so can instil greater pro‐environmental intentions across ideological divides.

## GENERAL DISCUSSION

Without significant intervention, current actions contributing to climate change will jeopardize the ecological well‐being of future generations. However, endeavours to combat climate change are often hindered by intense political polarization in many countries, impeding progress on this urgent societal issue. Consequently, inquiry into whether pro‐environmentalism can be promoted through less politically polarizing methods becomes increasingly crucial. Across 13 studies, we found that emphasizing the welfare of future generations, an issue less affected by partisan divisions compared to concern for climate change, holds promise for encouraging pro‐environmental behaviour. Across the United States (in various samples including scientists, community members, undergraduates and crowdsourced participants) and across 34 European countries, we observe widespread acknowledgement of responsibility to future generations (RFG). Notably, RFG surpasses perceived responsibility for mitigating personal contributions to climate change (RCC). Interestingly, Americans also believe others share this sentiment and view caring for future generations in the broad sense as morally superior to addressing climate change in particular.

Moreover, RFG is largely uncorrelated or weakly correlated with age, gender, race, income level, religiosity and education attainment. The only consistent correlations were with socioeconomic status, where across the 34 European countries a small positive correlation was observed, and in the United States, with political ideology. Importantly, across our many samples, the correlation between RFG and political ideology ranged from non‐significant (in Europe and some US samples) to moderate. However, in every single study, this correlation was significantly weaker than the correlation between RCC and political ideology, with the latter often having twice the magnitude. This suggests RFG is less politically polarized than RCC and holds significance in light of previous efforts to promote pro‐environmental attitudes having mainly focused on RCC, which often aligns with political leanings. In contrast, attention to RFG has only recently gained traction in research (e.g. Shrum et al., 2023; Syropoulos & Markowitz, 2023; Wade‐Benzoni, 2019). These findings, especially when coupled with recent insights on interventions targeting RFG (see Vlasceanu et al., 2024), are particularly encouraging because individuals with stronger RFG also reported greater engagement in pro‐environmental behaviours. This trend held true across diverse samples, political ideologies and pro‐environmental outcomes. Moreover, higher RFG correlated with reduced perceived psychological distance from, heightened perceptions of harm owing to and increased levels of worry about and commitment to mitigating climate change. These results are further supported by our experimental findings.

In Study 4, we replicated existing RFG interventions where participants reflected on their legacy, wrote letters to future generations or considered sacrifices made by past generations. Additionally, we tested two novel interventions, the first of which presented participants with text outlining the principles of longtermism (MacAskill, 2022). Among other ethical principles (e.g. utilitarian resource allocations), longtermism emphasizes moral obligation to positively impact the long‐term future by taking action in the present. Exposure to these principles increased RFG and self‐reported pro‐environmental attitudes, even despite climate change not being specifically mentioned in the text. The second novel intervention involved presenting participants with a 1‐min video emphasizing the importance of protecting nature for future generations. We chose short videos for their greater external validity compared to writing tasks, as they align more with people's daily behaviours. Additionally, both the longtermism and video conditions omitted writing, potentially facilitating scalability by focusing solely on content consumption rather than active task engagement.

An additional limitation concerns the strength of the correlation between RFG and RCC. Consistently in our studies, regardless of how these constructs were measured, correlations between them were strong. In fact, in exploratory analyses (see Figure S6 in SOM), in which RCC is treated as a sequential mediator following RFG, RCC explains the effect of RFG on pro‐environmentalism. At first glance, the strong correlation between these measures is troubling. However, several factors lessen this concern. First, the high correlation could be the product of common method variance, as in all studies we strove to keep the wording/verbiage of these items identical, with the exception being the target (future generations vs. climate change). Thus, this high correlation could be the product of the high amount of similarity in the language. Second, if these constructs were indeed the same mechanism, then a similar pattern of correlations with demographic variables would be noted. However, findings from Studies 1 and 2A‐2B suggest that there are notable differences in how these constructs correlate with demographic characteristics. For instance, RFG relates to political ideology and religiosity more weakly (and significantly so) than RCC.

Similarly (and as hypothesized), RFG is endorsed significantly more by Independents and Republicans than RCC, while RCC is endorsed at a similar amount as RFG for Democrats. Some experimental conditions, namely the condition replicating work by Watkins and Goodwin (2020), only increased RFG and not RCC, suggesting that these two types of responsibility are not always activated together. Furthermore, exploratory factor analysis (see Table S24 in SOM) supports treating these two constructs as different from each other/two factors. Ultimately, we also contend that climate change is an inherently intergenerational issue. Extant work has consistently noted that feeling responsible for protecting future generations is a robust predictor of pro‐environmentalism (Grolleau et al., 2021; Law et al., 2023; Shrum, 2021; Syropoulos et al., 2020; Syropoulos, Law, Mah, et al., 2024; Syropoulos, Law, & Young, 2024; Syropoulos & Markowitz, 2021; Vlasceanu et al., 2024; Watkins & Goodwin, 2020; Wickersham et al., 2020; Zaval et al., 2015). Thus, it is natural for people who care about future generations to also care about climate change. Nevertheless, future research should examine whether the strong correlation between these constructs varies in other cultural and national contexts and whether there are larger differences in the predictors and outcomes of these types of felt responsibility.

Our findings replicated the efficacy of the three existing interventions and indicated that the two new interventions were also effective. While every intervention increased pro‐environmental intentions and RFG, the letter writing condition emerged as potentially most effective. This conclusion was supported by both the breadth (i.e. all five outcomes) and magnitude of the effects. Moreover, each intervention had a significant indirect effect on all three pro‐environmental outcomes, with RFG positively predicting each one. Thus, our findings suggest that intergenerational approaches for motivating pro‐environmental engagement appear to have a small but consistent positive effect on pro‐environmental outcomes. Future work can extend these findings in five meaningful ways. First, longitudinal interventions can determine the enduring nature of these effects, and their application to everyday sustainability behaviours (e.g. taking the bus instead of driving to work, purchasing electric rather than fuel‐burning vehicles). Second, educational interventions with a focus on younger populations are needed to determine if enhancing longtermist and intergenerational values more broadly can have meaningful implications for one's beliefs, career choices and other consequential decisions later in life. From our preliminary evidence, it seems that reading about intergenerational values has a small positive influence. However, extensive longitudinal work is needed to determine whether deeper engagement with intergenerational values over time can leave a more lasting impact.

Third, additional evidence on how RFG relates to different moral values and virtues could further reveal its pervasive and depoliticized nature. Fourth, it is important to discover whether enhancing or making salient concerns for future generations also translates into greater support for and action towards addressing other (intergenerational) issues beyond climate change, such as inequality, poverty and core extinction threats discussed in popular, scientific and philosophical discourse (e.g. advanced AI, nuclear war and pandemics; MacAskill, 2022). Finally, drawing upon the moral reframing literature (e.g. Feinberg & Willer, 2019), future research should explore whether repackaging climate‐related concerns in intergenerational terms can increase pro‐environmental engagement among populations resistant to traditional rhetoric on climate change (e.g. climate change deniers and people on the far‐right). This approach may offer a promising avenue for overcoming barriers to environmental action and fostering broader support for sustainability initiatives even in nations and demographics where these issues are subject to robust political polarization.

### Limitations

This investigation was not without limitations. First, our evidence speaks solely to WEIRD populations (Henrich et al., 2010). Thus, extending this work beyond the Western context would meaningfully elucidate the universality of intergenerational beliefs. Initial evidence from a many‐labs global study lends credence to our results, finding evidence for the effectiveness of intergenerational interventions (Vlasceanu et al., 2024). Nevertheless, it is important to determine whether, for instance, observed effects may be amplified in nations with more collectivistic (Triandis, 1995), culturally tight (Gelfand et al., 2006) and long‐term oriented (Hofstede & Bond, 1984) cultures, as such societies emphasize the collective survival and well‐being of their members and exhibit stronger inclinations towards future preparation. Secondly, most measures were self‐reported. While we experimentally manipulated RFG and found support for a causal relationship with self‐reports of pro‐environmentalism, the impact on behavioural outcomes (i.e. donations) was small. Future research leveraging interlaboratory collaborations can augment self‐reports with more practical outcomes such as life‐cycle assessments and measures of greenhouse gas emissions linked to specific practices (e.g. Nielsen et al., 2022). Alternatively, employing newer behavioural paradigms like the Work for Environmental Protection Task (WEPT; Lange & Dewitte, 2022) can address more tangible outcomes. Finally, understanding individuals' perceptions of ‘future generations’ is essential. Do they envision their own descendants or a broader future population? How distant do they consider this future to be? While our studies do not directly explore these questions, forthcoming qualitative research could provide valuable insights, helping tailor language for intergenerational pro‐environmental approaches to resonate better with specific demographics. At first glance, research suggests that even intergenerational concern for distant future generations can be successfully manipulated via targeted interventions (Syropoulos, Law, Mah, et al., 2024; Syropoulos, Law, & Young, 2024).

## CONCLUSION

Addressing climate change stands as one of society's most critical challenges. Yet, political polarization surrounding related discourse often hinders meaningful individual and collective action against this looming threat. We provide initial evidence that emphasizing our responsibility to safeguard future generations could redirect the narrative away from the polarized discourse surrounding climate change towards actionable steps we can take today to ensure a more prosperous tomorrow. We contend that these findings, in tandem with emerging evidence that intergenerational framing interventions are relatively effective across political (Berkebile‐Weinberg et al., 2024) and cultural (Goldwert et al., 2024) divides, elucidate the potential usefulness of these interventions.

## AUTHOR CONTRIBUTIONS

**Stylianos Syropoulos:** Conceptualization; investigation; writing – original draft; methodology; validation; visualization; writing – review and editing; software; formal analysis; project administration; data curation. **Kyle Fiore Law:** Writing – original draft; conceptualization; writing – review and editing; visualization. **Gordon Kraft‐Todd:** Writing – review and editing; resources; funding acquisition. **Andrea Mah:** Data curation; writing – review and editing. **Ezra Markowitz:** Writing – review and editing. **Liane Young:** Supervision; writing – review and editing; resources; funding acquisition.

## CONFLICT OF INTEREST STATEMENT

The authors have no conflicts of interest to declare.

## Supporting information


Data S1.

## Data Availability

The data that support the findings of this study are openly available in Open Science Framework at https://osf.io/8s6zw/.

## Footnotes

^1^
Replicating findings from Studies 2A‐2B, Republicans expressed greater RFG compared to RCC, *t*(49) = 5.84, *p* < .001, *d* = 0.75, as did Independents, *t*(125) = 2.77, *p* = .006, *d* = 0.17. Democrats, conversely, did not differ in their perceptions of responsibility, *t*(208) = −1.89, *p* = .060.

^2^
This is also expressed in the raw frequency of participants who expressed responsibility to future generations over responsibility to reduce climate change (See Table S17 in SOM).

^3^
Importantly, when examining these associations specifically for each political group, the same pattern of results emerges (see Table S18 in SOM). Similarly, this pattern was also noted in a forced choice paradigm (see Figure S5 in SOM).

^4^
In fact, even when we present participants with a forced choice between protecting future generations or reducing climate change, a similar pattern of results were observed (see Supplementary Materials).

^5^
We did not include RCC as a mediator, due to concerns for collinearity with responsibility to future generations, *r* = .75, *p* < .001. However, we did consider a model in which this type of responsibility is a sequential mediator after RFG. In this model RFG still predicted two out of the three outcomes even after accounting for the effect of RCC (see Supplementary Materials).

## References

[bjso12775-bib-0001] Alper, B. A. (2022). How religion intersects with Americans' views on the environment . Pew Research Center's Religion & Public Life Project. https://www.pewresearch.org/religion/2022/11/17/how‐religion‐intersects‐with‐americans‐views‐on‐the‐environment/

[bjso12775-bib-0002] Berkebile‐Weinberg, M. , Goldwert, D. , Doell, K. C. , van Bavel, J. J. , & Vlasceanu, M. (2024). The differential impact of climate interventions along the political divide in 60 countries. Nature Communications, 15, 3885. 10.1038/s41467-024-48112-8 PMC11078920 38719845

[bjso12775-bib-0003] Brick, C. , Sherman, K. , & Kim, H. S. (2017). “Green to be seen” and “brown to keep down”: Visibility moderates the effect of identity on proenvironmental behavior. Journal of Environmental Psychology, 51, 226–238. 10.1016/j.jenvp.2017.04.004

[bjso12775-bib-0004] Crimston, C. R. , Bain, P. G. , Hornsey, M. J. , & Bastian, B. (2016). Moral expansiveness: Examining variability in the extension of the moral world. Journal of Personality and Social Psychology, 111(4), 636–653. 10.1037/pspp0000086 26751743

[bjso12775-bib-0005] Cruz, S. M. (2017). The relationships of political ideology and party affiliation with environmental concern: A meta‐analysis. Journal of Environmental Psychology, 53, 81–91. 10.1016/j.jenvp.2017.06.010

[bjso12775-bib-0006] Feinberg, M. , & Willer, R. (2019). Moral reframing: A technique for effective and persuasive communication across political divides. Social and Personality Psychology Compass, 13(12), e12501. 10.1111/spc3.12501

[bjso12775-bib-0007] Fox, M. , Tost, L. , & Wade‐Benzoni, K. (2010). The legacy motive: A catalyst for sustainable decision making in organizations. Business Ethics Quarterly, 20(2), 153–185. 10.5840/beq201020214

[bjso12775-bib-0008] Funk, C. (2021). Key findings: How Americans' attitudes about climate change differ by generation, party and other factors . Pew Research Center. https://www.pewresearch.org/fact‐tank/2021/05/26/key‐findings‐how‐americans‐attitudes‐about‐climate‐change‐differ‐by‐generation‐party‐and‐other‐factors/

[bjso12775-bib-0009] Geiger, N. , McLaughlin, B. , & Velez, J. (2021). Not all boomers: Temporal orientation explains inter‐ and intra‐cultural variability in the link between age and climate engagement. Climatic Change, 166(1), 1–20. 10.1007/s10584-021-03116-x

[bjso12775-bib-0010] Gelfand, M. J. , Nishii, L. H. , & Raver, J. L. (2006). On the nature and importance of cultural tightness‐looseness. Journal of Applied Psychology, 91(6), 1225–1244. 10.1037/0021-9010.91.6.1225 17100480

[bjso12775-bib-0011] Gifford, R. (2011). The dragons of inaction: Psychological barriers that limit climate change mitigation and adaptation. American Psychologist, 66(4), 290–302. 10.1037/a0023566 21553954

[bjso12775-bib-0012] Goh, J. X. , Hall, J. A. , & Rosenthal, R. (2016). Mini meta‐analysis of your own studies: Some arguments on why and a primer on how. Social and Personality Psychology Compass, 10(10), 535–549. 10.1111/spc3.12267

[bjso12775-bib-0013] Goldwert, D. , Bao, Y. E. , Doell, K. C. , Van Bavel, J. J. , & Vlasceanu, M. (2024). The effects of climate action interventions along cultural individualism‐collectivism. 10.31234/osf.io/cv3n4

[bjso12775-bib-0014] Gonzalez‐Ricoy, E. , & Rey, F. (2019). Enfranchising the future: Climate justice and the representation of future generations. WIRE Climate Change, 10(5), e598. 10.1002/wcc.598

[bjso12775-bib-0016] Graham, J. , Waytz, A. , Meindl, P. , Iyer, R. , & Young, L. (2017). Centripetal and centrifugal forces in the moral circle: Competing constraints on moral learning. Cognition, 167, 58–65. 10.1016/j.cognition.2016.12.001 28007293

[bjso12775-bib-0017] Grandin, A. , Guillou, L. , Sater, R. A. , Foucault, M. , & Chevallier, C. (2022). Socioeconomic status, time preferences and proenvironmentalism. Journal of Environmental Psychology, 79, 101720. 10.1016/j.jenvp.2021.101720

[bjso12775-bib-0018] Grolleau, G. , Mzoughi, N. , Napoleone, C. , & Pellegrin, C. (2021). Does activating legacy concerns make farmers more likely to support conservation programmes? Journal of Environmental Economics and Policy, 10(2), 1–15. 10.1080/21606544.2020.1807410

[bjso12775-bib-0019] Haushofer, J. , & Fehr, E. (2014). On the psychology of poverty. Science, 344(6186), 862–867. 10.1126/science.1232491 24855262

[bjso12775-bib-0020] Helferich, M. , Thorgensen, J. , & Berguist, M. (2023). Direct and mediated impacts of social norms on proenvironmental behavior. Global Environmental Change, 80, 102680. 10.1016/j.gloenvcha.2023.102680

[bjso12775-bib-0021] Henrich, J. , Heine, S. J. , & Norenzayan, A. (2010). The weirdest people in the world? Behavioral and Brain Sciences, 33(2–3), 61–83. 10.1017/S0140525X0999152X 20550733

[bjso12775-bib-0022] Hofstede, G. , & Bond, M. H. (1984). Hofstede's culture dimensions: An independent validation using Rokeach's value survey. Journal of Cross‐Cultural Psychology, 15(4), 417–433. 10.1177/0022002184015004003

[bjso12775-bib-0023] Howe, P. , Mildenberger, M. , Marlon, J. , & Leiserowitz, A. (2015). Geographic variation in opinions on climate change at state and local scales in the USA. Nature Climate Change, 5, 596–603. 10.1038/nclimate2583

[bjso12775-bib-0024] Hurlstone, M. J. , Price, A. , Wang, S. , Leviston, Z. , & Walker, I. (2020). Activating the legacy motive mitigates intergenerational discounting in the climate game. Global Environmental Change, 60, 102008. 10.1016/j.gloenvcha.2019.102008

[bjso12775-bib-0025] IPCC . (2022). Climate change widespread, rapid, and intensifying – IPCC . https://www.ipcc.ch/2021/08/09/ar6‐wg1‐20210809‐pr/

[bjso12775-bib-0026] Jamieson, D. (2015). Responsibility and climate change. Global Justice: Theory Practice Rhetoric, 8(2), 23–42. 10.21248/gjn.8.2.86

[bjso12775-bib-0027] Joireman, J. A. , Van Lange, P. A. M. , & Van Vugt, M. (2004). Who cares about the environmental impact of cars?: Those with an eye toward the future. Environment and Behavior, 36(2), 187–206. 10.1177/0013916503251476

[bjso12775-bib-0028] Kennedy, B. (2020). U.S. concern about climate change is rising, but mainly among democrats . Pew Research Center. https://www.pewresearch.org/fact‐tank/2020/04/16/u‐s‐concern‐about‐climate‐change‐is‐rising‐but‐mainly‐among‐democrats/

[bjso12775-bib-0069] Kline, R. B. (2015). Principles and Practice of Structural Equation Modeling (4th ed.). The Guilford Press.

[bjso12775-bib-0029] Klockner, C. A. (2013). A comprehensive model of the psychology of environmental behaviour—A meta‐analysis. Global Environmental Change, 23(5), 1028–1038. 10.1016/j.gloenvcha.2013.05.014

[bjso12775-bib-0030] Lange, F. , & Dewitte, S. (2022). The work for environmental protection task: A consequential web‐based procedure for studying proenvironmental behavior. Behavior Research Methods, 54, 133–145. 10.3758/s13428-021-01617-2 34109560

[bjso12775-bib-0031] Law, K. F. , Syropoulos, S. , & Young, L. (2023). Why do longtermists care about protecting the environment? An investigation into the underlying mechanisms of pro‐climate policy support. Sustainability, 15(24), 16732. 10.3390/su152416732

[bjso12775-bib-0032] Litman, L. , Robinson, J. , & Abberbock, T. (2017). TurkPrime.Com: A versatile crowdsourcing data acquisition platform for the behavioral sciences. Behavior Research Methods, 49(2), 433–442. 10.3758/s13428-016-0727-z 27071389 PMC5405057

[bjso12775-bib-0033] MacAskill, W. (2022). What we owe the future. Basic Books.

[bjso12775-bib-0035] Martinez, E. , & Winter, C. (2023). The intuitive appeal of legal protection for future generations (SSRN Scholarly Paper 4349899). https://papers.ssrn.com/abstract–4349899

[bjso12775-bib-0036] McManus, R. M. , Kleiman‐Weiner, M. , & Young, L. (2020). What we owe to family: The impact of special obligations on moral judgment. Psychological Science, 31(3), 227–242. 10.1177/0956797619900321 31990627

[bjso12775-bib-0037] Milfont, T. L. , & Duckitt, J. (2010). The environmental attitudes inventory: A valid and reliable measure to assess the structure of environmental attitudes. Journal of Environmental Psychology, 30(1), 80–94. 10.1016/j.jenvp.2009.09.001

[bjso12775-bib-0038] Nielsen, K. S. , Brick, C. , Hofmann Joanes, T. , Lange, F. , & Gwozdz, W. (2022). The motivation–impact gap in proenvironmental clothing consumption. Nature Sustainability, 5, 665–668. 10.1038/s41893-022-00888-7

[bjso12775-bib-0039] Ord, T. (2020). The precipice: Existential risk and the future of humanity. Hachette Books.

[bjso12775-bib-0040] Oreskes, N. , & Conway, E. M. (2010). Merchants of doubt: How a handful of scientists obscured the truth on issues from tobacco smoke to global warming. Bloomsbury Press.

[bjso12775-bib-0041] Park, H. S. , Ulusoy, E. , Choi, S. Y. , & Lee, H. E. (2020). Temporal distance and descriptive norms on environmental behaviors: A cross‐cultural examination of construal‐level theory. SAGE Open, 10(1), 2158244020914576. 10.1177/2158244020914576

[bjso12775-bib-0042] Rottman, J. , Kelemen, D. , & Young, L. (2015). Hindering harm and preserving purity: How can moral psychology save the planet? Philosophy Compass, 10(2), 134–144.

[bjso12775-bib-0043] Schwartz, S. H. (1977). Normative influences on altruism. In L. Berkowitz (Ed.), Advances in experimental social psychology (Vol. 10 (pp. 221–279). Academic Press.

[bjso12775-bib-0044] Schwartz, S. H. , & Howard, J. A. (1981). A normative decision‐making model of altruism. In J. P. Rushton & R. M. Sorrentino (Eds.), Altruism and helping behavior (pp. 189–211). Lawrence Erlbaum.

[bjso12775-bib-0045] Shrum, T. R. (2021). The salience of future impacts and the willingness to pay for climate change mitigation: An experiment in intergenerational framing. Climatic Change, 165, 18. 10.1007/s10584-021-03002-6

[bjso12775-bib-0046] Shrum, T. R. , Platt, N. , Syropoulos, S. , & Markowitz, E. (2023). A review of the green parenthood effect on environmental and climate engagement. WIRE Climate Change, 14(2), e818. 10.1002/wcc.818

[bjso12775-bib-0047] Soliman, M. , Alisat, S. , Bashir, N. Y. , & Wilson, A. E. (2018). Wrinkles in time and drops in the bucket: Circumventing temporal and social barriers to pro‐environmental behavior. SAGE Open, 8(2), 2158244018774826. 10.1177/2158244018774826

[bjso12775-bib-0048] Stern, P. C. (2000). Toward a coherent theory of environmentally significant behavior. Journal of Social Issues, 56(3), 407–424. 10.1111/0022-4537.00175

[bjso12775-bib-0049] Stern, P. C. , Dietz, T. , Abel, T. D. , Guagnano, G. A. , & Kalof, L. (1999). A value‐belief norm theory of support for social movements: The case of environmentalism. Research in Human Ecology, 6(2), 81–97.

[bjso12775-bib-0050] Syropoulos, S. , Law, K. F. , Mah, A. , & Young, L. (2024). Intergenerational concern relates to constructive coping and emotional reactions to climate change via increased legacy concerns and environmental cognitive alternatives. BMC Psychology, 12, 182. 10.1186/s40359-024-01690-0 38566114 PMC10986099

[bjso12775-bib-0051] Syropoulos, S. , Law, K. F. , & Young, L. (2024). Longtermist education interventions increase concern for and action to protect future generations. Social Psychological and Personality. Science. 19485506241228465. 10.1177/19485506241228465

[bjso12775-bib-0052] Syropoulos, S. , & Markowitz, E. (2021). Perceived responsibility towards future generations and environmental concern: Convergent evidence across multiple outcomes in a large and nationally representative sample. Journal of Environmental Psychology, 76, 101651. 10.1016/j.jenvp.2021.101651

[bjso12775-bib-0053] Syropoulos, S. , & Markowitz, E. (2022). Perceived responsibility consistently relates to increased proenvironmental attitudes, behaviors and policy support: Evidence across 23 countries. Journal of Environmental Psychology, 83, 101868. 10.1016/j.jenvp.2022.101868

[bjso12775-bib-0054] Syropoulos, S. , & Markowitz, E. (2024). Responsibility towards the future is a strong predictor of proenvironmental engagement. Journal of Environmental Psychology, 93, 102218. 10.1016/j.jenvp.2023.102218

[bjso12775-bib-0055] Syropoulos, S. , & Markowitz, M. (2023). Our responsibility to future generations: The case for intergenerational approaches to the study of climate change. Journal of Environmental Psychology, 87, 102006. 10.1016/j.jenvp.2023.102006

[bjso12775-bib-0056] Syropoulos, S. , Watkins, H. , Shariff, A. , Hodges, S. , & Markowitz, E. (2021). The role of gratitude in motivating intergenerational environmental stewardship. Journal of Environmental Psychology, 72, 101517. 10.1016/j.jenvp.2020.101517

[bjso12775-bib-0057] Triandis, H. C. (1995). Individualism & collectivism. Westview Press.

[bjso12775-bib-0058] Tyson, A. , Funk, C. , & Kennedy, B. (2023). What the data says about Americans' views of climate change . Pew Research Center. https://www.pewresearch.org/short‐reads/2023/08/09/what‐the‐data‐says‐about‐americans‐views‐of‐climate‐change/

[bjso12775-bib-0059] van Valkengoed, A. M. , Steg, L. , & Perlaviciute, G. (2023). The psychological distance of climate change is overestimated. One Earth, 6(4), 362–391. 10.1016/j.oneear.2023.03.006

[bjso12775-bib-0060] Vlasceanu, M. , Doell, K. C. , Bak‐Coleman, J. B. , Todorova, B. , Berkebile‐Weinberg, M. M. , Grayson, S. J. , Patel, Y. , Goldwert, D. , Pei, Y. , Chakroff, A. , Pronizius, E. , van den Broek, K. , Vlasceanu, D. , Constantino, S. , Morais, M. J. , Schumann, P. , Rathje, S. , Fang, K. , Aglioti, S. M. , … van Bavel, J. (2024). Addressing climate change with behavioral science: A global intervention tournament in 63 countries. Science Advances, 10, eadj5778. 10.1126/sciadv.adj5778 38324680 PMC10849597

[bjso12775-bib-0062] Wade‐Benzoni, K. A. (2019). Legacy motivations & the psychology of intergenerational decisions. Current Opinion in Psychology, 26, 19–22. 10.1016/j.copsyc.2018.03.013 29709868

[bjso12775-bib-0063] Wade‐Benzoni, K. A. , & Tost, L. P. (2009). The egoism and altruism of intergenerational behavior. Personality and Social Psychology Review, 13(3), 165–193. 10.1177/1088868309339317 19571118

[bjso12775-bib-0064] Wang, Y. , Hao, F. , & Liu, Y. (2021). Proenvironmental behavior in an aging world: Evidence from 31 countries. International Journal of Environmental Resources and Public Health, 18(4), 1748. 10.3390/ijerph18041748 PMC7916887 33670167

[bjso12775-bib-0065] Watkins, H. M. , & Goodwin, G. P. (2020). Reflecting on sacrifices made by past generations increases a sense of obligation towards future generations. Personality and Social Psychology Bulletin, 46(7), 995–1012. 10.1177/0146167219883610 31743077

[bjso12775-bib-0068] Wickersham, R. H. , Zaval, L. , Pachana, N. A. , & Smyer, M. A. (2020). The impact of place and legacy framing on climate action: A lifespan approach. PLOS ONE, 15(2), e0228963. 10.1371/journal.pone.0228963 32097411 PMC7041806

[bjso12775-bib-0066] Xiao, C. , & McCright, A. M. (2013). Gender differences in environmental concern: Revisiting the institutional trust hypothesis in the USA. Environment and Behavior, 47(1), 17–37. 10.1177/0013916513491571

[bjso12775-bib-0067] Zaval, L. , Markowitz, E. M. , & Weber, E. U. (2015). How will I Be remembered? Conserving the environment for the sake of One's legacy. Psychological Science, 26(2), 231–236. 10.1177/0956797614561266 25560825

