# Supplementary material for: Responsibility to future generations: A strategy for combatting climate change across political divides
Source: Br J Soc Psychol. 2024 Jun 20;64(1):e12775. doi: 10.1111/bjso.12775 (PMC11590069; doi:10.1111/bjso.12775)
Supplement: Supplementary file 1 — Data S1. [file BJSO-64-0-s001.docx]

Responsibility to Future Generations: A Strategy for Combatting Climate Change Across Political Divides

**Supplementary Materials**

**Table of Contents**

Study 1 – Supplementary Information ………………………………………………........ page 3

Studies S1A-S1B ………………………………………………......................................... page 5

Study S2 ………………………………………………...................................................... page 12

Study S3A ……………………………………………………............................................ page 16

Study S3B ……………………………………………………............................................ page 18

Study 2B – Supplementary Information ..……………………………………………........ page 21

Study S4 ……...………………………………………........................................................ page 22

Study 3A – Supplementary Information ..……………………………………………........ page 30

Study S5 ……...………………………………………........................................................ page 35

Study 3B – Supplementary Information ..……………………………………………........ page 41

Study 4 – Supplementary Information ..……………………………………………........... page 44

Analyses Requested by Reviewers ..……………………………………………........... page 49

References ..…………………………………………….................................................... page 52

**Study 1 – Supplementary Information**

The number of participants interviewed in each survey, for each country is provided in Table S1. Further, associations within each country are reported in Table S2.

**Table S1**

*Number of Participants Per Country for each Eurobarometer Survey*

| Country | 2009 | 2010a | 2010b | 2011 | Total |
| --- | --- | --- | --- | --- | --- |
| France | 900 | 917 | 1000 | 989 | 3806 |
| Belgium | 970 | 988 | 990 | 1013 | 3961 |
| The Netherlands | 964 | 962 | 1011 | 994 | 3931 |
| Germany West | 952 | 962 | 1010 | 975 | 3899 |
| Italy | 924 | 902 | 965 | 996 | 3787 |
| Luxembourg | 461 | 478 | 504 | 489 | 1932 |
| Denmark | 939 | 963 | 1029 | 996 | 3927 |
| Ireland | 787 | 847 | 921 | 961 | 3516 |
| Great Britain | 900 | 888 | 905 | 942 | 3635 |
| Northern Ireland | 258 | 250 | 269 | 274 | 1051 |
| Greece | 978 | 968 | 983 | 983 | 3912 |
| Spain | 927 | 928 | 938 | 976 | 3769 |
| Portugal | 846 | 890 | 982 | 1020 | 3738 |
| Germany East | 483 | 474 | 530 | 502 | 1989 |
| Finland | 978 | 955 | 986 | 995 | 3914 |
| Sweden | 1000 | 1012 | 998 | 1026 | 4036 |
| Austria | 960 | 932 | 959 | 979 | 3830 |
| Cyprus (Republic) | 454 | 454 | 488 | 494 | 1890 |
| Czech Republic | 987 | 971 | 973 | 983 | 3914 |
| Estonia | 918 | 914 | 935 | 953 | 3720 |
| Hungary | 982 | 978 | 987 | 1000 | 3947 |
| Latvia | 920 | 924 | 958 | 977 | 3779 |
| Lithuania | 866 | 889 | 888 | 949 | 3592 |
| Malta | 393 | 406 | 420 | 449 | 1668 |
| Poland | 851 | 852 | 905 | 900 | 3508 |
| Slovakia | 982 | 961 | 999 | 975 | 3917 |
| Slovenia | 958 | 955 | 959 | 995 | 3867 |
| Bulgaria | 827 | 860 | 938 | 951 | 3576 |
| Romania | 834 | 870 | 906 | 948 | 3558 |
| Turkey | 809 | 784 | 25336 | 823 | 27752 |
| Croatia | 888 | 917 | 0 | 945 | 2750 |
| FYROM | 876 | 964 | 0 | 982 | 2822 |
| Montenegro | 0 | 0 | 0 | 959 | 959 |
| Iceland | 0 | 500 | 0 | 497 | 997 |
| Total | 26772 | 27515 | 25336 | 29890 | 109513 |

**Table S2**

*Correlations of Each Demographic Indicator With Responsibility to Future Generations in Each Country*

| Country | Political Ideology | Socioeconomic Status | Age | Gender (female = 1) |
| --- | --- | --- | --- | --- |
| France | **0.17** | **0.18** | 0.08 | -0.05 |
| Belgium | -0.01 | 0.08 | 0.03 | -0.04 |
| Netherlands | -0.04 | **0.17** | **0.16** | **-0.10** |
| Germany West | -0.02 | **0.13** | 0.06 | -0.06 |
| Italy | 0.06 | **0.10** | 0.02 | -0.02 |
| Luxembourg | 0.02 | **0.13** | 0.15 | -0.03 |
| Denmark | 0.02 | **0.13** | -0.01 | -0.04 |
| Ireland | 0.01 | 0.07 | 0.09 | -0.03 |
| Great Britain | 0.03 | 0.08 | 0.02 | -0.02 |
| Northern Ireland | 0.00 | 0.00 | 0.05 | -0.06 |
| Greece | **0.10** | **0.14** | 0.02 | 0.00 |
| Spain | 0.02 | 0.08 | -0.02 | -0.01 |
| Portugal | 0.00 | 0.03 | -0.03 | -0.02 |
| Germany East | -0.03 | **0.10** | -0.01 | -0.07 |
| Finland | 0.05 | 0.08 | -0.01 | -0.03 |
| Sweden | -0.04 | 0.08 | 0.04 | -0.01 |
| Austria | 0.01 | **0.13** | -0.03 | 0.02 |
| Cyprus (Republic) | -0.01 | -0.01 | 0.10 | -0.06 |
| Czech Republic | **0.22** | **0.17** | -0.06 | -0.02 |
| Estonia | **0.14** | **0.14** | -0.03 | -0.08 |
| Hungary | 0.04 | **0.11** | -0.02 | -0.02 |
| Latvia | 0.04 | 0.06 | 0.06 | -0.03 |
| Lithuania | **0.11** | 0.08 | -0.05 | -0.01 |
| Malta | **0.14** | **0.10** | 0.05 | 0.00 |
| Poland | 0.00 | 0.05 | -0.02 | -0.04 |
| Slovakia | **0.18** | **0.21** | -0.05 | -0.02 |
| Slovenia | -0.05 | **0.14** | -0.01 | -0.02 |
| Bulgaria | 0.09 | **0.11** | 0.00 | -0.02 |
| Romania | 0.00 | 0.02 | 0.04 | -0.01 |
| Turkey | 0.03 | 0.03 | 0.02 | -0.02 |
| Croatia | 0.02 | 0.05 | 0.05 | -0.03 |
| Macedonia | 0.01 | **0.10** | 0.04 | -0.01 |
| Montenegro | -0.07 | -0.02 | 0.07 | -0.04 |
| Iceland | **-0.14** | 0.09 | **0.11** | 0.02 |

**Note**. Bolded correlations are significant at *p* < .001.

**Studies S1A-S1B**

Two supplementary studies further examined the prevalence of how much people think about future generations within the context of sustainability and the environment. In these two surveys the items relevant to concern for future generations focused specifically on whether people thought that damage to the environment could pose a risk to future generations. Two Eurobarometer surveys conducted 20 years apart (each with different samples) from several European countries were examined. These studies were not pre-registered.

**Methods**

***Participants***

**Study S1A (Eurobarometer 29, 1988).** The 29^th^ Eurobarometer survey was conducted in 12 different European countries (see Table S3 for a list of these countries). A total of 11,729 participants were surveyed. The average age was 42 years (SD = 18.12). Gender was roughly evenly split, with 6,084 women (52%) and 5,629 men (48%) participating in the survey.

**Study S1B (Eurobarometer 69, 2008).** The 69^th^ Eurobarometer survey was conducted in 31 different European countries (see Table S4 for a list of these countries). A total of 30,170 participants were surveyed. The average age was 47 years (SD = 18.19). In this sample, 16,420 were women (55%) and 13,750 were men (45%).

***Measures***

**Study S1A.** Of relevance to the current study was the measure that captured concern for future generations. In the interviews conducted as part of the Eurobarometer survey, a topic which was discussed, was damage to the environment. As part of that conversation the interviewers noted whether participants referenced that “damage already made to the environment risks having consequences for future generations”. This was coded as 1 = reference was made, 0 = reference was not made.

**Study S1B.** Similarly to Study S1A, as part of the interview for the Eurobarometer survey, the interviewers asked participants the following question: “There are many reasons why people take actions aimed at fighting climate change. Please tell me which of the following apply to you?”. Then several options were given, with participants being given the opportunity to select multiple options. Of interest to the current investigation was the option concerning future generations “You are very concerned about the world that you will leave for the young and future generations.” Responses were marked as 1 = mentioned, and 0 = not mentioned.

**Results**

All analyses were conducted in SAS version 9.4. We estimated the frequency of each response by using the *proc freq* command, while sorting by each country.

***Study S1A –* *1988* *Eurobarometer***

As seen in Figure S1, the percentage of participants who expressed concern for the well-being of future generations when considering the potential risk produced by damage to the environment ranged between 25%-70%. These results suggested that at least 1 in 4 people across 12 countries expressed concern for future generations. Crucially this was an unprompted response, as participants referenced this future generations on their own accord.

**Table S3**

*Percentage of Participants who Expressed that “damage already made to the environment risks having consequences for future generations” in Study 1A (Eurobarometer 29, 1988)*

| Country | Percentage |
| --- | --- |
| Portugal | 25% |
| Greece | 27% |
| Ireland | 30% |
| Belgium | 38% |
| Spain | 40% |
| Germany | 41% |
| United Kingdom | 41% |
| Italy | 43% |
| Luxembourg | 47% |
| France | 50% |
| Netherlands | 53% |
| Denmark | 70% |

**Figure S1**

*Map of the Countries Included in Study 1A Displaying the Range of Percentages of Participants who Expressed Concern for Future Generations in 1988*


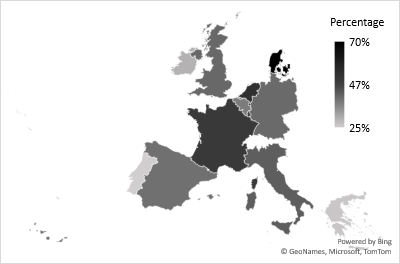


***Study S1B –* *2008* *Eurobarometer***

In the 2008 Eurobarometer the measure of concern for future generations was explicitly framed in the context of climate change. In this survey, the percentage of participants who were concerned for future generations when referencing the reasons for taking action towards climate change ranged from 7% to 58% (see Figure S2). However, a large number of participants stated that they took no action against climate change (*M* = 43%, *Median* = 37% across all countries). When examining the percentage of participants who expressed concern for future generations as a one of the reasons why they took action against climate change specifically, this percentage increases from a minimum of 31% to a maximum of 79% (see Figure S3).

**Figure S2**

*Map of the Countries Included in Study 1B Displaying the Range of Percentages of Participants who Expressed Concern for Future Generations in 2008*


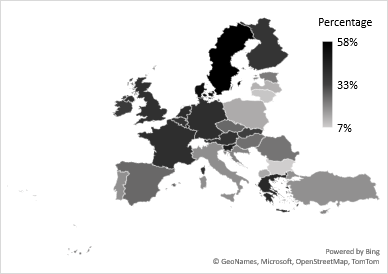


**Figure S3**

*Map of the Countries Included in Study 1B Displaying the Range of Percentages of Participants who Expressed Concern for Future Generations as a Reason They Took Action to Address Climate Change Relative to All Participants Who Took Action Against Climate Change in 2008*


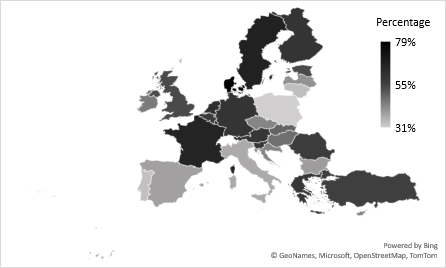


**Table S4**

*Percentage of Participants Who Expressed that they Are “very concerned about the world that you will leave for the young and future generations” when thinking about actions aimed to fight climate change in Study 1B (Eurobarometer 69, 2008)*

| Country | Percentage relative to  all participants | Percentage relative to those who take action against climate change |
| --- | --- | --- |
| Bulgaria | 7% | 40% |
| Lithuania | 9% | 34% |
| Latvia | 12% | 41% |
| Poland | 13% | 31% |
| Northern Macedonia | 14% | 45% |
| Portugal | 18% | 34% |
| Turkey | 18% | 56% |
| Italy | 19% | 37% |
| Cyprus | 20% | 44% |
| Croatia | 21% | 44% |
| Estonia | 22% | 54% |
| Romania | 23% | 57% |
| Hungary | 24% | 47% |
| Spain | 25% | 39% |
| Czech Republic | 26% | 43% |
| Malta | 29% | 44% |
| Slovakia | 33% | 49% |
| Ireland | 35% | 45% |
| Finland | 37% | 60% |
| Belgium | 37% | 57% |
| Austria | 38% | 59% |
| United Kingdom | 45% | 59% |
| France | 40% | 66% |
| Germany | 40% | 60% |
| Netherlands | 41% | 61% |
| Greece | 42% | 59% |
| Slovenia | 46% | 58% |
| Denmark | 53% | 79% |
| Luxembourg | 53% | 66% |
| Sweden | 58% | 67% |

**Note.** For Cyprus one sample was obtained for Greek Cypriots and one for the Turkish population and their average was estimated. For Germany one sample was collected in Eastern and one in Western Germany and their average was estimated. For the United Kingdom we averaged samples from Great Britain and Northern Ireland.

**Discussion**

Studies S1A and S1B used data from interviews conducted in Europe in the last 35 years obtained from two Eurobarometers. Their purpose was to examine the degree to which people consider future generations a reason to act proenvironmentally. Overall, we found in one survey that at least one in 4 (Study S1A) and that roughly between 1 in 10 (Study 1B) participants express such concerns. Importantly, when we consider those who are environmentally conscious, this percentage rises to at least 4 in 10. These descriptive results suggest that even 35 years ago, concerns for future generations were salient. Thus, although descriptive, such evidence suggests that protecting future generations could be an important motivator for engaging in proenvironmental action.

**Study S2**

In this second supplementary study we sought to re-examine the findings of Studies 1A-1D and Studies S1A-S1B. However, this time the group of interest were experts/scientists. Through a survey of the Ecological Society of American we were able to get insight on how much ecologists in the U.S. valued protecting future generations.

**Methods**

***Participants***

A total of 1215 participants completed the survey. From this sample, 817 were male, and 396 were female, and 2 did not provide a response. The majority of the members had a PhD/MD or equivalent degree (*n* = 1020, 84%). Age was captured in ranges, with the average age being indicative of 40-49 years.

***Measures***

Participants completed a long survey about their attitudes and beliefs. Of relevance to the current investigation was the following question: “The next set of questions is meant to learn what attributes and values you think an ecologist ought to possess…” Responses were captured on a 0-100 slider scale, with the following labels 10 = harmful, 30 = risky, 50 = irrelevant, 70 = helpful, 90 = vital. A total of 14 responses were available, and of interest was the one focusing on future generations “has a sense of duty to future generations.” Additional measures focusing on different forms of proenvironmental attitudes, beliefs and behaviors were included. Associations between these measures and reported duty to future generations are reported in Table S3.

**Results**

Overall the average sense of duty to future generations was 68.71 (SD = 16.89). Importantly, only 3.9% considered one’s duty to future generations as harmful or risky, 22% considered it irrelevant, with the remaining 74.1% considered it at least relevant (see Figure S4 for the distribution of the scores).

**Figure S4**

*Frequency of scores for ESA members’ sense of duty to future generations.*


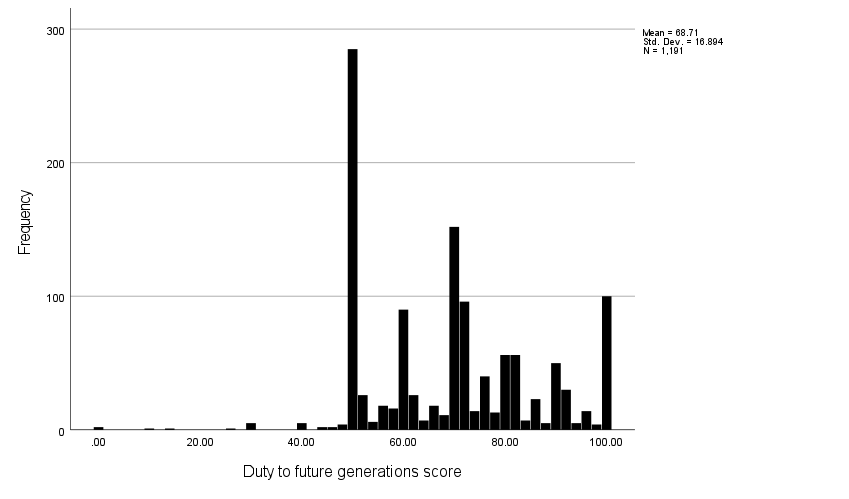


**Table S5**

*Relevant Information for Variables Included in Study S2*

| Item | Correlation with Duty to Future Generations (*r*) | Range | Labels |
| --- | --- | --- | --- |
| How often do you provide financial support for an environmental cause as a private citizen? | **0.10*** | 1-6 | 1 = never, 2 = less than once per year, 3 = 1-2 times per year, 4 = 3-6 times per year, 5 = 7-12 times per year, 6 = more than 12 times per year |
| Approximately how much money do you donate to environmental causes each year? | **0.11*** | 1-7 | 1 = $0, 2 = $1-$99, 3 = $100-$249, 4 = $250-$499, 5 = $500-$999, 6 = $1000-$5000, 7 = more than $5000 |
| How important is it to you to engage in some personal environmental action each day (such as bicycling to work, recycling paper, or buying green products)? | **0.13*** | 1-7 | 1 = Not at all important, 7 = Very important |
| Approximately how often do you write a letters to an editor, politician, or agency concerning an environmental cause, as a self-identified professional ecologist? | **0.17*** | 1-6 | 1 = never, 2 = less than once per year, 3 = 1-2 times per year, 4 = 3-6 times per year, 5 = 7-12 times per year, 6 = more than 12 times per year |
| How often do you speak publicly concerning an environmental cause, as a private citizen without referring to your ecological expertise? | **0.20*** | 1-6 | 1 = never, 2 = less than once per year, 3 = 1-2 times per year, 4 = 3-6 times per year, 5 = 7-12 times per year, 6 = more than 12 times per year |
| How often have you provided organizational leadership for or against an environmental cause, such as prohibiting beach development? | **0.17*** | 1-4 | 1 = never, 2 = once, 3 = 2-3 times,  4 = 4 or more times |
| Has an appreciation of the beauty of nature | **0.44*** | 0 - 100 | 0 = harmful, 30 = risky, 50 = irrelevant,  70 = helpful, 90 = vital |
| Believes that nature is intrinsically valuable | **0.49*** | 0 - 100 | 0 = harmful, 30 = risky, 50 = irrelevant,  70 = helpful, 90 = vital |
| Believes that humans have caused serious damage to the natural world | **0.42*** | 0 - 100 | 0 = harmful, 30 = risky, 50 = irrelevant,  70 = helpful, 90 = vital |
| Believes that we are morally obligated to preserve aspects of the natural world | **0.55*** | 0 - 100 | 0 = harmful, 30 = risky, 50 = irrelevant,  70 = helpful, 90 = vital |
| Enjoys being outdoors | **0.36*** | 0 - 100 | 0 = harmful, 30 = risky, 50 = irrelevant,  70 = helpful, 90 = vital |
| Seeks to be of service to society | **0.62*** | 0 - 100 | 0 = harmful, 30 = risky, 50 = irrelevant,  70 = helpful, 90 = vital |
| Values knowledge for its own sake | **0.16*** | 0 - 100 | 0 = harmful, 30 = risky, 50 = irrelevant,  70 = helpful, 90 = vital |
| Has a personal affinity for the entity, process, or place he/she studies | **0.36*** | 0 - 100 | 0 = harmful, 30 = risky, 50 = irrelevant,  70 = helpful, 90 = vital |

**Note.** **p* < .001.

**Discussion**

Study S2 illustrated that much like the general public, experts also share a sense of duty to future generations. In a sample of ecologists, a sense of duty to future generations was considered relevant for the majority (3/4 of the sample). Further, 38.1% considered it helpful, and 13.8% vital. Further those who thought that having a sense of duty to future generations is an important value for an ecologist, also were also more likely to endorse other important values (e.g., appreciating beauty, valuing nature, preserving nature) and scored higher on self-reports of proenvironmental behaviors, a finding which will be replicated with more precise measures in later studies in this investigation.

**Study S3A**

Study S3A served as a direct replication of Study 2A. Our goal was to re-examine responsibility for future generations would be endorsed to a greater degree than responsibility to reduce climate change in a relatively younger sample of American participants who strongly believed in anthropogenic climate change.

**Methods**

Data was collected from a large, public research university in the greater New England area in the Fall 2022 semester. Participants were students who took part in a large screening survey which included various measures. In this survey, we were able to include our measures of interest and conducted our analyses solely with these measures.

***Participants***

A total of 632 students completed the survey. The average age was 19.61 years (SD = 1.74). The majority of the sample was female (*n* = 500, 79.24%), with 127 male students (20.13%) and the rest either declined to answer or indicated some other category. The majority of the sample was white (*n* = 389, 62.04%), 134 students were Asian (21.37%) 23 (3.67%) were Black, 20 (3.19%) were Latinx/Hispanic, and the rest were multiracial (*n* = 60, roughly 10%). Almost every student in the sample (*n* = 601, 98%) believed that climate change was at the very least mainly caused by human activity.

***Measures***

**Perceived responsibility*.*** To capture perceived responsibility to reduce climate change, we used an item taken from large publicly available surveys (“To what extent do you feel a personal responsibility to try to reduce climate change?”; e.g., Syropoulos & Markowitz, 2022). To measure perceived responsibility towards future generations we used the four items used by Syropoulos and colleagues (2020), and a single item taken from publicly available surveys (e.g., “To what degree is the following value important to you as a reason for protecting the environment? - Living up to our responsibility to protect future generations.”; Syropoulos & Markowitz, 2021). One item which was reverse coded (“My generation needs to look after itself first and worry about future generations second.”) was removed due to poor factor loading. The resulting 4-item construct had good reliability (*a* = .80).

**Results**

***Students Feel More Responsible towards Future Generations***

Responsibility to reduce climate change and responsibility towards future generations were strongly correlated, *r* = .52, *p* < .001. Responsibility towards future generations (*M* = 5.14, *SD* = 1.06) was endorsed significantly more, *t*(616) = 7.55, *p* < .001, *d* = 0.30, than responsibility to reduce climate change (*M* = 4.76, *SD* = 1.41). This was also the case for students who expressed less belief in the anthropogenic causes of climate change (reflected by only agreeing that climate change is mainly caused by humans but not entirely so), *t*(426) = 6.10, *p* < .001, *d* = 0.29; responsibility towards future generations: *M* = 5.07, *SD* = 1.03; responsibility to reduce climate change: *M* = 4.72, *SD* = 1.35.

Descriptively, 211 students (34.20%) endorsed responsibility to reduce climate change more than responsibility towards future generations; 67 students (10.86%) endorsed both equally; the remaining 339 students (54.94%) expressed more responsibility towards future generations relative to responsibility to reduce climate change.

**Discussion**

Study S3A provided a conceptual replication of the results obtained from Study 2. Importantly, these results emerged when we measured responsibility to future generations with additional items, in a sample of younger adults that showed a high degree of belief in climate change.

**Study S3B**

This study provided a replication of Studies 2A-2B and Study S3A in a sample obtained from a large public university. Our goal was to further establish whether even in a relatively young sample, participants would feel more responsible to future generations than they would relative to their responsibility to reduce climate change. Further, we sought to use nearly-identically worded measures for both types of responsibility to reduce any alternative explanations for this effect.

**Methods**

Data were again collected from a large, public research university in the greater New England area in the Spring 2023 semester. Participants were students who took part in a large screening survey which included various measures. In this survey, we were able to include our measures of interest and conducted our analyses solely with these measures.

***Participants***

A total of 762 students completed the survey. The average age was 19.68 years (SD = 1.53). The majority of the sample was female (*n* = 576, 75.69%), with 181 male students (23.78%) and the rest either declined to answer or indicated some other category. The majority of the sample was white (*n* = 493, 65.00%), 151 students were Asian (19.89%) 23 (3.03%) were Black, 27 (3.56%) were Latinx/Hispanic, and the rest were multiracial (*n* = 68, roughly 9%). Almost every student in the sample (*n* = 698, 94.32%) believed that climate change was at the very least mainly caused by human activity.

***Measures***

**Perceived responsibility*.*** To capture perceived responsibility to reduce climate change, and responsibility to future generations we used the same four items, with the only difference being the target of the statement. Items for future generations are given as an example: (1) When deciding how to live, I have a duty to consider the impact of my actions on future generations, (2) When we make decisions today we should take into consideration how our actions influence future generations, (3) I’m willing to sacrifice aspects of my own life if it will help people living in the future, (4) People living today have an obligation to protect future generations. Both 4-item constructs were captured on a 1-7 Likert scale (1 = strongly disagree – 7 = strongly agree) and had good reliability (*a*s > .83). Finally, we also used the same measure of belief in anthropogenic climate change as Study S3A.

**Results**

***Students Feel More Responsible towards Future Generations***

An unrestricted exploratory factor analysis suggested that two factors are observed, such that responsibility to future generations and responsibility to reduce climate change are distinct factors, with each factor having an eigen value > 3. Replicating the findings of Study S3A, our analyses suggested that responsibility to reduce climate change and responsibility towards future generations were strongly correlated, *r* = .54, *p* < .001. Responsibility towards future generations (*M* = 5.36, *SD* = 0.91) was endorsed significantly more, *t*(751) = 5.71, *p* < .001, *d* = 0.21, than responsibility to reduce climate change (*M* = 5.16, *SD* = 1.07).

This was also the case for students who expressed less belief in the anthropogenic causes of climate change, *t*(54) = 4.23, *p* < .001, *d* = 0.57; responsibility towards future generations: *M* = 4.94, *SD* = 0.94; responsibility to reduce climate change: *M* = 4.15, *SD* = 1.29, and those who did, *t*(696) = 4.48, *p* < .001, *d* = 0.17; responsibility towards future generations: *M* = 5.39, *SD* = 0.90; responsibility to reduce climate change: *M* = 5.24, *SD* = 1.01.

Descriptively, 269 students (35.77%) endorsed responsibility to reduce climate change more than responsibility towards future generations; 119 students (15.82%) endorsed both equally and the remaining 364 students (48.40%) expressed more responsibility towards future generations relative to responsibility to reduce climate change.

**Discussion**

Even when we use nearly identically-worded measures of responsibility we were able to replicate the difference between how responsible people feel to future generations compared to the issue of climate change. Importantly, this effect emerges in a sample comprising younger participants, who tend to believe in climate change.

**Study 2B – Supplementary Information**

***Accuracy of Perceived Prevalence***

Recent evidence (Sparkman et al., 2022) suggests that Americans have a false perception of the social reality concerning climate change, such that they underestimate how much support there is for policies that seek to address the issue. Such inaccurate perceptions can demotivate individuals from engaging in meaningful action to resolve issues such as climate change. Considering this, we examined whether a participant’s own average score would be closer to the perceived prevalence of each type of responsibility. We estimated this score by subtracting the score for perceived prevalence (descriptive norms) from the actual score of each type of responsibility. We then subjected these scores to a paired sample t-test to determine whether the difference is statistically significant. We found that for responsibility to future generations (*M* = 9.03, *SD* = 27.24), the average score of participants was significantly closer to the perceived prevalence of this type of responsibility, relative to responsibility to address climate change (*M* = 11.48, *SD* = 26.62), *t*(905) = -2.65, *p* = .008, *d* = -0.09, although notably this effect was very small.

**Study S4**

In this study we sought to examine how responsibility to future generations relates to a host of proenvironmental outcomes. Since the measure used was only a proxy for responsibility to future generations, as it conflated responsibility to future generations with responsibility to protect the environment, we considered these results supplementary to those reported in Studies 3A-3B. Further, we were able to examine whether responsibility to future generations was not significantly correlated with different demographic indicators replicating the findings of Study 1 in the United States. This study was pre-registered.

**Methods**

***Participants***

The survey was conducted in the greater Detroit area in 2002. Data were accessed via the Inter-University Consortium for Political and Social Research (ICPSR 24320 Detroit Area Study, 2002). A total of 328 participants completed the measures of interest. In this sample, 198 were male, 133 were female, and 3 did not provide a response. More than half of the sample was white (*n* = 181), with the other 40% being Black or African American (*n* = 132). The rest was Asian or Asian American, some other identity, or did not provide a response. The average age was 48.40 years (*SD* = 16.89).

***Materials***

**Responsibility to future generations.** Participants were asked to express their agreement or disagreement to the following statement “We have to protect the environment for

future generations, even if it means reducing our present level of consumption.” Scores were captured on an 1-4 Likert scale, ranging from 1= strongly disagree to 4 = strongly agree.

**Proenvironmental attitudes, beliefs and behaviors.** A total of 39 items was implemented to examine people’s attitudes towards the environment. These ranged from concern about different environmental issues and the environment in general, attitudes towards nature and animal welfare/justice, proenvironmental beliefs, intentions and proenvironmental behaviors. These are summarized in detail in Table S6. Measures that were thematically similar, and on the same measurement scale, were subjected to exploratory factor analysis, followed by a reliability test. Based on these tests were meaningfully aggregated these measures into unitary constructs (see Table S5 for further information).

**Demographic indicators.** All demographic indicators were measured with a single item each. Education levels ranged from 1 (less than high school) to 6 (graduate or professional degree). Income ranged from 1 (less than $10m000) to 9 ($125,000 or more). Political ideology ranged from 1 (extremely liberal) to 7 (extremely conservative). Two aspects of religiosity were measured. How important religion was to the person (1-4 scale, 1 = not important at all, 4 = very important), and how frequently they attended religious services (1 = a few times a year, 4 = at least once a week).

**Exploratory Items.** One item, (“I believe that protecting nature is important for religious or spiritual reasons.”) was used to examine whether responsibility to future generations was endorsed more as a reason to protect the environment. This item was included in the survey and was the only one of its kind. Importantly, it was also captured on the same scale as the item focusing on responsibility to future generations. We hypothesized, that on average, responsibility to future generations would be endorsed significantly more.

In addition, an item focusing on anger towards past generations with regards to how much they have damaged the planet (“The young ought to be angry at previous generations over what they have done to the planet”). This item too was captured on a 1-4 scale (1 = strongly disagree – 4 = strongly agree). We hypothesized that more anger towards past generations would relate to increased responsibility towards future generations.

**Table S6**

*Information for All Proenvironmental Outcomes Included in Study S4*

| Number | Item | ICPSR Survey Identifier | Scale | Lowest label | Highest Label |
| --- | --- | --- | --- | --- | --- |
| 1 | Ranking of “improving and protecting the environment” relative to: education, economic equality between blacks and whites, protecting the nation economy, reducing crime rate, and solving the drug problem | B18d | 0-6 | 0 (lowest ranking) | 6 (highest ranking) |
| 2 | How personally concerned are you about environmental issues? | C3 | 1-7 | not at all concerned | Extremely concerned |
| 3 | Which one of these statements about the environment and the economy comes closest to your own view? | C4 | 0-1 | Economic growth should be given priority, even if the environment suffers to some extent. | Protecting the environment should be given priority, even at the risk of slowing down economic growth. |
| 4 | How personally concerned are you about environmental problems facing this country | C6 | 1-4 | not at all concerned | Very concerned |
| 5 | How personally concerned are you about environmental problems facing the world as a whole? | C8 | 1-4 | not at all concerned | Very concerned |
| 6 | How personally concerned are you about environmental problems facing your neighborhood? | C10 | 1-4 | not at all concerned | Very concerned |
| 7 | Do you consider yourself to be an environmentalist? | C15 | 0-1 | No | Yes |
| 8 | How often do you keep thermostats turned down in winter to save energy? | E1a | 1-5 | Never | Always |
| 9 | How often do you have broken items fixed instead of buying new ones? | E1b | 1-5 | Never | Always |
| 10 | How often do you turn off lights to save electricity? | E1c | 1-5 | Never | Always |
| 11 | How often do you take steps to conserve gasoline? For example, by using public transportation, walking, or car pooling? | E1d | 1-5 | Never | Always |
| 12 | How often do you choose to buy food products that are organically grown- that is, without pesticides or chemicals? | E1e | 1-5 | Never | Always |
| 13 | How often do you save materials for recycling? | E1f | 1-5 | Never | Always |
| 14 | How often do you buy some product specifically because you think it is better for the environment than competing products? | E1g | 1-5 | Never | Always |
| 15 | How serious of a problem is... the pollution of drinking water? | F1a | 1-4 | Not a problem at all | Very serious problem |
| 16 | How serious of a problem is...the loss of natural scenic areas? | F1b | 1-4 | Not a problem at all | Very serious problem |
| 17 | How serious of a problem is... the depletion of the ozone layer? | F1c | 1-4 | Not a problem at all | Very serious problem |
| 18 | How serious of a problem are…shortages of energy to run our cars, homes and businesses? | F1d | 1-4 | Not a problem at all | Very serious problem |
| 19 | How serious of a problem are... oil spills? | F1e | 1-4 | Not a problem at all | Very serious problem |
| 20 | How serious of a problem is... safely getting rid of hazardous wastes? | F1f | 1-4 | Not a problem at all | Very serious problem |
| 21 | How serious of a problem is… global warming or the greenhouse effect? | F1g | 1-4 | Not a problem at all | Very serious problem |
| 22 | How serious of a problem are… shortages of water in this country for farms, homes, and businesses? | F1h | 1-4 | Not a problem at all | Very serious problem |
| 23 | How serious of a problem is... air pollution? | F1i | 1-4 | Not a problem at all | Very serious problem |
| 24 | How serious of a problem is... the loss of natural places for fish and wildlife to live? | F1j | 1-4 | Not a problem at all | Very serious problem |
| 25 | How serious of a problem is... too much waste and garbage? | F1k | 1-4 | Not a problem at all | Very serious problem |
| 26 | How serious of a problem is... acid rain? | F1l | 1-4 | Not a problem at all | Very serious problem |
| 27 | How serious of a problem is... pesticides and chemicals in food? | F1m | 1-4 | Not a problem at all | Very serious problem |
| 28 | How serious of a problem is... the loss of rain forests and jungles? | F1n | 1-4 | Not a problem at all | Very serious problem |
| 29 | How willing would you be to pay higher taxes to the government if you knew the money would be spent to protect the environment and prevent land, water and air pollution? | F3 | 1-4 | Not willing at all | Very willing |
| 30 | How willing would you be to work with other people in your community to improve the environment by spending two hours a week working on environmental projects? | F4 | 1-4 | Not willing at all | Very willing |
| 31 | How willing would you be to work with other people in your community to improve the environment by contributing money? | F5 | 1-4 | Not willing at all | Very willing |
| 32 | How much do you personally worry about "global warming" or the "greenhouse effect?" | F7 | 1-4 | Not at all | A great deal |
| 33 | Living in a clean, healthy, and safe environment is a human right. | G1 | 1-4 | Strongly disagree | Strongly agree |
| 34 | It should be guaranteed by law that land and resources are used in ways that ensure there will be enough for everyone, now and in the future. | G4 | 1-4 | Strongly disagree | Strongly agree |
| 35 | Here are three statements people sometimes make when discussing the relationship between human beings and nature. Which one of them comes closest to your own point of view? | H2 | 0-1 | Nature exists primarily to be used by humans. | Nature is sacred and should be left alone or Humans must live in harmony with nature in order to survive. |
| 36 | Every plant and animal species has value of its own, even if we don't have a human use for it | H3f | 1-4 | Strongly disagree | Strongly agree |
| 37 | Justice is not just for human beings, we need to be as fair to plants and animals as we are towards people | H3i | 1-4 | Strongly disagree | Strongly agree |
| 38 | Nature is inherently beautiful. When we see ugliness in the environment it is caused by humans. | H3k | 1-4 | Strongly disagree | Strongly agree |
| 39 | What would you choose in the following situation… A developer wants to build a mall which will create new jobs on land that has been declared environmentally sensitive by a local environmental organization. How much would you support the decision to let the developer build the mall? | H11 | 1-4 | Strongly oppose | Strongly support |

**Table S7**

*Constructs Created based on Exploratory Factor Analyses and Reliability tests, with Items for Each Construct Found via the “number” Column of Table S6*

| Construct | Number of items | Items from Table S6 | EFA Factor Loadings range | Reliability |
| --- | --- | --- | --- | --- |
| General Environmental Concern | 3 | 4-6 | .50 -.75 | .72 |
| Proenvironmental Behaviors | 7 | 8-14 | .40 - .63 | .69 |
| Environmental Problem Risk Perception | 14 | 16-28 | .58 - .74 | .91 |
| Proenvironmental Intentions | 3 | 29-31 | .57 - .70 | .71 |

**Note.** For each EFA, eigen values were > 1.

**Results**

In line with our pre-registration, we used the weight command to include the weights (W_NORMAL) generated by the original research team.

***Association of Responsibility to Future Generations with Demographic Indicators***

Responsibility to future generations was not significantly correlated with age (*r* = -.04, *p* = .500), income (*r* = .10, *p* = .084), education level (*r* = .04, *p* = .493), importance of religion (*r* = .04, *p* = .479) and attendance to religious practices (*r* = .06, *p* = .307). However, a small negative correlation with political ideology was observed (*r* = -.14, *p* = .010).

***Responsibility to Future Generations and Proenvironmental Outcomes***

Responsibility to future generations related to all proenvironmental outcomes positively and significantly, except for the belief that nature is sacred. These associations were robust to the inclusion of all demographic indicators as covariates in the analysis. R square values ranged from .05 to .22, suggesting that responsibility to future generations explains at least 5% of the variance in proenvironmental outcomes.

**Table S8**

*Linear and Logistic Regressions (Indicated by a † Symbol) for Each Outcome. Model 1 Includes Only Responsibility to Future Generations as a predictor; Model 2 Also Includes Age, Gender (Male = 1), Income, Education, Political Ideology, Religious importance and Attendance to Religious Services as Covariates*

| Outcome | Model 1 | | Model 2 | |
| --- | --- | --- | --- | --- |
|  | β (SE) | Adj R^2^ (OR) | β (SE) | Adj R^2^ (OR) |
| Ranking protecting the environment as a top priority | **.23*** (.14)** | 0.05 | **.28*** (.17)** | 0.10 |
| How personally concerned are you about environmental issues? | **.26*** (.13)** | 0.07 | **.25*** (.15)** | 0.10 |
| Protecting the environment should be given priority, even at the risk of slowing down economic growth. † | **.20** (.19)** | (1.76) | **.25** (.23)** | 2.15 |
| Do you consider yourself to be an environmentalist? † | **.41*** (.28)** | (3.20) | **.41*** (.32)** | 3.58 |
| How much do you personally worry about "global warming" or the "greenhouse effect?" | **.30*** (.08)** | 0.08 | **.29*** (.09)** | 0.11 |
| Living in a clean, healthy, and safe environment is a human right. | **.25*** (.06)** | 0.06 | **.19** (.06)** | 0.19 |
| It should be guaranteed by law that land and resources are used in ways that ensure there will be enough for everyone, now and in the future. | **.27*** (0.07)** | 0.07 | **22*** (.07)** | 0.16 |
| Nature is sacred and should be left alone or Humans must live in harmony with nature in order to survive. † | .16 (.31) | (1.56) | .16 (.37) | 1.65 |
| Every plant and animal species has value of its own, even if we don't have a human use for it | **.29*** (.06)** | 0.08 | **.28*** (.07)** | 0.05 |
| Justice is not just for human beings, we need to be as fair to plants and animals as we are towards people | **.25*** (.08)** | 0.06 | **.19** (.09)** | 0.25 |
| Nature is inherently beautiful. When we see ugliness in the environment it is caused by humans. | **.30*** (.07)** | 0.09 | **.25*** (.08)** | 0.11 |
| A developer wants to build a mall which will create new jobs on land that has been declared environmentally sensitive by a local environmental organization. How much would you support the decision to let the developer build the mall? | **-.23*** (.07)** | 0.05 | **-.20** (.09)** | 0.03 |
| General Environmental Concern | **.28*** (.05)** | 0.07 | **.32*** (.06)** | 0.15 |
| Proenvironmental Behaviors | **.22*** (.06)** | 0.05 | **.19** (.07)** | 0.10 |
| Environmental Problem Risk Perception | **.32*** (.04)** | 0.10 | **.28*** (.05)** | 0.18 |
| Proenvironmental Intentions | **.47*** (.06)** | 0.22 | **.35*** (.07)** | 0.29 |

**Note.** Significant results are bolded. Adj = Adjusted. OR = Odds Ratio. ** *p* < .01, *** *p* < .001.

***Pre-registered Exploratory Analyses***

We pre-registered two exploratory analyses. The first sought to determine whether people would endorse protecting the environment for future generations significantly more compared to protecting the environment for religious or spiritual reasons. Indeed, a paired sample t-test supported this hypothesis, indicating that people expressed greater responsibility to future generations (*M* = 3.45, *SD* = 0.64) compared to religion or spirituality (*M* = 2.73, *SD* = 0.95) as a reason why they would protect the environment, *t*(327) = 12.31, SE = .06, *p* < .001, *d* = 0.889.

The second exploratory test sought to examine whether perceptions of anger over past generations damage to the environment would relate to increased responsibility to future generations (with regards to protecting the environment). Indeed, a significant positive association was noted, *r* = .30, *p* < .001, suggesting that anger towards past generations related to an increased sense of responsibility to future generations.

**Discussion**

Overall, our fourth supplementary study served as a companion to Studies 1, 2A-2B, 3A-3B. It did so by replicating the associations between demographic indicators and responsibility to future generations, which were for the most part not significant. Further, it illustrated that those who felt responsible to future generations scored higher on a host of proenvironmental outcomes, explaining a non-negligible amount of variance in each case. Further, the two exploratory analyses also expand on the usefulness of this moral reframing mechanism, by suggesting that people are more likely to protect the environment for future generations rather than for their own religion or spirituality, and by noting a positive association between anger for damages by past generation and a willingness to help future generations as a result. Importantly, even though the sample in this study was considerably smaller than that of each other study, the uniqueness of the sample (i.e., a U.S. community sample) makes it more diverse than the typical student or MΤurk sample, offering a pre-registered replication of our results in a different community setting.

**Study 3A – Supplementary Information**

**Correlations for Individual Items**

Similar results were observed between the demographic indicators and each individual item of both responsibility constructs. In a similar vein, identical results were observed for associations between the proenvironmental outcomes and each individual item of both responsibility constructs.

**Table S9**

*Bivariate Correlations between All Items of the Two Responsibility Measures and Demographic Indicators*

|  | When deciding how to live, I have a duty to consider the impact of my actions on ***future generations*** | When we make decisions today we should take into consideration how our actions influence ***future generations*** | I’m willing to sacrifice aspects of my own life if it will help ***people living in the future*** | People living today have an obligation to protect ***future generations*** | I feel a personal responsibility to do my best to protect ***future generations*** |
| --- | --- | --- | --- | --- | --- |
| Ideology | **-0.27***** | **-0.32***** | **-0.24***** | **-0.29***** | **-0.19***** |
| Dem-Rep | **-0.24***** | **-0.29***** | **-0.15**** | **-0.24***** | **-0.17***** |
| Income | 0.07 | 0.03 | 0.07 | 0.10* | 0.09 |
| Religion | 0.05 | -0.07 | 0.00 | -0.06 | 0.05 |
| Age | 0.04 | -0.03 | -0.05 | 0.06 | 0.06 |
| Education | 0.09 | 0.04 | 0.05 | **0.11*** | 0.06 |
|  | When deciding how to live, I have a duty to consider the impact of my actions on ***climate change*** | When we make decisions today we should take into consideration how our actions contribute to ***climate change*** | I’m willing to sacrifice aspects of my own life if it will help reduce the negative impacts of ***climate change*** | People living today have an obligation to reduce ***climate change*** | I feel a personal responsibility to do my best to reduce ***climate change*** |
| Ideology | **-0.46***** | **-0.51***** | **-0.44***** | **-0.46***** | **-0.43***** |
| Dem-Rep | **-0.43***** | **-0.48***** | **-0.38***** | **-0.45***** | **-0.41***** |
| Income | 0.01 | 0.02 | 0.00 | 0.05 | 0.02 |
| Religion | -0.11* | -0.20*** | **-0.12*** | **-0.14**** | -0.09 |
| Age | -0.02 | -0.05 | -0.08 | -0.04 | -0.02 |
| Education | **0.12*** | **0.11*** | **0.11*** | **0.10*** | **0.13**** |

**Note.** * *p* < .05, ** *p* < .01, *** *p* < .001. Dem-Rep = Democrat-Republican continuum, a measure which ranged from 1 – 7.

**Table S10**

*Bivariate Correlations between All Items of the Two Responsibility Measures and Proenvironmental Outcomes*

|  | When deciding how to live, I have a duty to consider the impact of my actions on ***future generations*** | When we make decisions today we should take into consideration how our actions influence ***future generations*** | I’m willing to sacrifice aspects of my own life if it will help ***people living in the future*** | People living today have an obligation to protect ***future generations*** | I feel a personal responsibility to do my best to protect ***future generations*** |
| --- | --- | --- | --- | --- | --- |
| CC Harm | **0.44*** | **0.45*** | **0.39*** | **0.45*** | **0.40*** |
| CC now | **-0.22*** | **-0.29*** | **-0.21*** | **-0.30*** | **-0.19*** |
| CC Concern | **0.46*** | **0.47*** | **0.38*** | **0.50*** | **0.44*** |
| PEBs | **0.43*** | **0.43*** | **0.37*** | **0.47*** | **0.44*** |
|  | When deciding how to live, I have a duty to consider the impact of my actions on ***climate change*** | When we make decisions today we should take into consideration how our actions contribute to ***climate change*** | I’m willing to sacrifice aspects of my own life if it will help reduce the negative impacts of ***climate change*** | People living today have an obligation to reduce ***climate change*** | I feel a personal responsibility to do my best to reduce ***climate change*** |
| CC Harm | **0.65*** | **0.67*** | **0.61*** | **0.63*** | **0.63*** |
| CC now | **-0.35*** | **-0.42*** | **-0.32*** | **-0.43*** | **-0.32*** |
| CC Concern | **0.72*** | **0.72*** | **0.64*** | **0.72*** | **0.70*** |
| PEBs | **0.55*** | **0.52*** | **0.51*** | **0.51*** | **0.58*** |

**Note.** * *p* < .001. Dem-Rep = Democrat-Republican continuum, a measure which ranged from 1 – 7. CC = Climate Change. PEB = proenvironmental behaviors.

**Perceptions of Harm For Specific Entities and Responsibility**

Highly similar associations in terms of significance, magnitude and directionality were observed for perceptions of harm towards all entities for both types of responsibility respectively.

**Table S11**

*Bivariate Correlations Between Both Types of Responsibility and Perceptions that Climate Change Will Harm Different Entities*

|  | …you personally | …your family | …your community | …people in the US | …future generations | …plant and animal species |
| --- | --- | --- | --- | --- | --- | --- |
| RFG | 0.39* | 0.42* | 0.41* | 0.43* | 0.43* | 0.46* |
| RCC | 0.53* | 0.55* | 0.56* | 0.61* | 0.70* | 0.70* |

**Note.** * *p* < .001.

**Comparisons of Responsibility for Each Item**

Overall highly similar results as our previous studies emerged for each individual item, suggesting that in general, Republicans and Independents tend to express greater responsibility to future generations relative to responsibility to reduce climate change.

**Table S12**

*Means, Standard Deviations, Paired-Sample t-tests and Effect Sizes for Each Individual Item of Responsibility*

|  | RFG | | RCC | |  |  |
| --- | --- | --- | --- | --- | --- | --- |
| All participants (N = 395) | M | SD | M | SD | t-test | Cohen's d |
| When deciding how to live, I have a duty to consider the impact of my actions on ***future generations***/ ***climate change*** | 5.41 | 1.28 | 5.20 | 1.50 | t(394) = 3.81, *p <* .001 | 0.15 |
| When we make decisions today we should take into consideration how our actions influence ***future generations***/ ***contribute to climate change*** | 5.80 | 1.06 | 5.54 | 1.36 | t(394) = 4.68, *p <* .001 | 0.21 |
| I’m willing to sacrifice aspects of my own life if it will help ***people living in the future***/ ***reduce the negative impacts of climate change*** | 5.06 | 1.36 | 4.98 | 1.49 | t(394) = 1.12, *p =* .264 | -- |
| People living today have an obligation to ***protect*** ***future generations***/ ***reduce climate change*** | 5.63 | 1.20 | 5.55 | 1.40 | t(394) = 1.52, *p =* .106 | -- |
| I feel a personal responsibility to do my best to protect ***future generations***/ ***reduce climate change*** | 5.34 | 1.30 | 5.15 | 1.51 | t(394) = 3.44, *p <* .001 | 0.13 |
| Republicans (N = 50) | M | SD | M | SD | t-test | Cohen's d |
| When deciding how to live, I have a duty to consider the impact of my actions on ***future generations***/ ***climate change*** | 4.94 | 1.28 | 3.92 | 1.81 | t(49) = 5.29,  *p <* .001 | 0.65 |
| When we make decisions today we should take into consideration how our actions influence ***future generations***/ ***contribute to climate change*** | 5.12 | 1.14 | 4.10 | 1.54 | t(49) = 4.93,  *p <* .001 | 0.75 |
| I’m willing to sacrifice aspects of my own life if it will help ***people living in the future***/ ***reduce the negative impacts of climate change*** | 4.60 | 1.47 | 3.58 | 1.63 | t(49) = 4.48,  *p <* .001 | 0.66 |
| People living today have an obligation to ***protect*** ***future generations***/ ***reduce climate change*** | 5.14 | 1.28 | 4.20 | 1.76 | t(49) = 4.94,  *p <* .001 | 0.61 |
| I feel a personal responsibility to do my best to protect ***future generations***/ ***reduce climate change*** | 4.96 | 1.31 | 3.86 | 1.70 | t(49) = 5.09,  *p <* .001 | 0.72 |
| Independents (N = 126) | M | SD | M | SD | t-test | Cohen's d |
| When deciding how to live, I have a duty to consider the impact of my actions on ***future generations***/ ***climate change*** | 5.28 | 1.39 | 5.04 | 1.59 | t(125) = 2.36, *p =* .020 | 0.16 |
| When we make decisions today we should take into consideration how our actions influence ***future generations***/ ***contribute to climate change*** | 5.75 | 1.03 | 5.44 | 1.35 | t(125) = 3.30, *p =* .001 | 0.26 |
| I’m willing to sacrifice aspects of my own life if it will help ***people living in the future***/ ***reduce the negative impacts of climate change*** | 5.06 | 1.36 | 4.97 | 1.56 | t(125) = 0.93, *p =* .353 | -- |
| People living today have an obligation to ***protect*** ***future generations***/ ***reduce climate change*** | 5.53 | 1.19 | 5.38 | 1.45 | t(125) = 1.50, *p =* .137 | -- |
| I feel a personal responsibility to do my best to protect ***future generations***/ ***reduce climate change*** | 5.33 | 1.37 | 5.01 | 1.55 | t(125) = 3.20, *p =* .001 | 0.22 |
| Democrats (N = 209) | M | SD | M | SD | t-test | Cohen's d |
| When deciding how to live, I have a duty to consider the impact of my actions on ***future generations***/ ***climate change*** | 5.58 | 1.19 | 5.59 | 1.14 | t(208) = -0.16, *p =* .870 | -- |
| When we make decisions today we should take into consideration how our actions influence ***future generations***/ ***contribute to climate change*** | 5.98 | 1.00 | 5.95 | 1.06 | t(208) = 0.42, *p =* .675 | -- |
| I’m willing to sacrifice aspects of my own life if it will help ***people living in the future***/ ***reduce the negative impacts of climate change*** | 5.13 | 1.33 | 5.32 | 1.20 | t(208) = -3.06, *p =* .002 | 0.15 |
| People living today have an obligation to ***protect*** ***future generations***/ ***reduce climate change*** | 5.81 | 1.17 | 5.94 | 1.04 | t(208) = -2.15, *p =* .033 | 0.12 |
| I feel a personal responsibility to do my best to protect ***future generations***/ ***reduce climate change*** | 5.42 | 1.27 | 5.51 | 1.25 | t(208) = -1.42, *p =* .156 | -- |

**RCC and Proenvironmental Outcomes**

Overall, with and without the addition of demographic covariates, greater responsibility to reduce climate change predicted increased engagement in proenvironmental behaviors, concern for climate change, perceptions of harm from climate change and that climate change will harm people in the US sooner rather than later in the future.

**Table S13**

*Linear Regression models With and Without Covariates for Responsibility to Reduce Climate Change as the Predictor.*

|  |  | RCC only | | | | |  |  | RCC and covariates | | | | | |
| --- | --- | --- | --- | --- | --- | --- | --- | --- | --- | --- | --- | --- | --- | --- |
| Outcome | b | β | SE | Lower C.I. | Upper C.I. | Adj. R^2^ |  | b | | β | SE | Lower C.I. | Upper C.I. | Adj. R^2^ |
| Proenvironmental behaviors frequency | 0.23*** | 0.56 | 0.02 | 0.19 | 0.26 | 0.31 |  | 0.21*** | | 0.53 | 0.02 | 0.17 | 0.25 | 0.33 |
| Concern for climate change | 0.89*** | 0.73 | 0.04 | 0.81 | 0.97 | 0.54 |  | 0.72*** | | 0.60 | 0.05 | 0.63 | 0.81 | 0.59 |
| Years in the future climate change will occur | -9.08*** | -0.40 | 1.06 | -11.16 | -7.00 | 0.16 |  | -5.60*** | | -0.25 | 1.15 | -7.87 | -3.33 | 0.25 |
| Climate change will harm people | 0.49*** | 0.65 | 0.03 | 0.43 | 0.54 | 0.43 |  | 0.39*** | | 0.53 | 0.03 | 0.33 | 0.45 | 0.50 |
| Donation per month (USD $) | -0.05 | -.00 | 2.69 | -5.34 | 5.25 | .00 |  | 6.01 | | 0.11 | 2.97 | 0.17 | 11.86 | 0.12 |

*Note.* Adj = Adjusted.

**Study S5**

This study aimed to test the research questions examined in Study 3B. However due to the order the questions in the survey were presented, significant order effects were detected which prevented us from testing our pre-registered hypotheses. Thus we conducted a second version of the study, for which we also sought to recruit more participants who identified as Independent and Republican, given a notably small number of Republican/right-leaning participants in this study. Results are largely consistent across studies despite this limitation.

**Methods**

***Participants***

A total of 561 participants were recruited via Prolific. Participants signed up for a study which lasted 12 minutes and received $2.50 as remuneration. One participant was removed due to failing an attention check. In this sample, 263 were female, and 280 male, with 13 indicating that they were non-binary or some other response option. The majority of the sample was White American (n = 405), and from the remaining participants, 60 were Black or African American, 58 were Asian or Asian American, and the rest indicated that they were indigenous, multiracial or some other racial identity. In terms of party identification, 286 were Democrats, 186 were Independents, 65 were Republicans, and the remaining 24 indicated some other category. The average age was 38 years (SD = 13.74).

***Measures***

Measures were shown to participants in the following groups, with measures within each group presented in a randomized order: (1) responsibility to future generations, (2) proenvironmental outcomes and responsibility to reduce climate change, (3) demographic variables. The order of the first two groups of measures (Group 1: responsibility to future generations, Group 2: proenvironmental outcomes and responsibility to future generations) was counter-balanced. We measured responsibility towards future generation with 2 items: “When deciding how to live, I have a duty to consider the impact of my actions on future generations” and “We need to reform to benefit future generations even if that means making some sacrifices now” (*a* = .93). Both items were captured on 7-point Likert scales ranging from 1 = strongly disagree to 7 = strongly agree. We measured responsibility to reduce greenhouse emissions (2 items, *a* = .87), carbon emissions (2 items, *a* = .87), and global warming (2 items, *a* = .89) using the same stem and Likert scale.

To measure proenvironmental behavioral engagement and attitudes we used several sub-scales from the environmental attitudes inventory created by Milfont and Duckitt (2010). We included the 6-item version of following sub-scales, nearly all of which were reliable (*a*s ranging from .83 to .90): (1) Interventionist Conservation Policies (*M* = 5.22, *SD* = 1.20), Environmental Threat (*M* = 5.54, *SD* = 1.20), Personal Conservation Behavior (*M* = 5.66, *SD* = 0.98), Conservation Motivated by Anthropocentric Concern (*M* = 3.41, *SD* = 0.91), Eccentric Concern (*M* = 6.01, *SD* = 0.91), and Support for population growth policies (*M* = 4.65, *SD* = 1.49). Importantly, the Conservation Motivated by Anthropocentric Concern sub-scale had mediocre reliable, *a* = .66). We had intended to also include the Environmental Movement Activism sub-scale, but due to experimental error the measure was not included in the study. Political ideology was measured with the same 2-items as Study 3A-3B (*a* = .90).

**Results**

Hypotheses for this study were pre-registered. However due to significant order effect (reported below) we were not able to test some of these hypotheses.

***Order Effects***

Unexpectedly, a significant order effect was observed for responsibility to reduce global warming (*F*(1, 558) = 5.61, *p* = .018, η^2^_p_ = .010), carbon emissions (*F*(1, 558) = 4.10, *p* = .043, η^2^_p_ = .007) and greenhouse emissions (*F*(1, 558) = 6.46, *p* = .011, η^2^_p_ = .011), such that each type of responsibility was higher when participants first completed the measure of responsibility to future generations (*N* = 260, global warming: *M* = 5.63, *SD =* 1.26, carbon emissions: *M* = 5.53, *SD =* 1.24, greenhouse emissions: *M* = 5.59, *SD =* 1.20) than when they completed this measure last (*N* = 300, global warming: *M* = 5.35, *SD =* 1.53, carbon emissions: *M* = 5.29, *SD =* 1.51, greenhouse emissions: *M* = 5.30, *SD =* 1.48). No significant effect on responsibility to future generations was observed (*F*(1, 558) = 3.57, *p* = .059). Considering this, and the relatively lower number of Republican participants in the study, we opted to not conduct any within-person comparisons between the different types of responsibility. No significant effect on any of the outcome measures capturing proenvironmental attitudes and behaviors was observed (all *p*s > .05). Notably, this order effect, although unexpected, suggests that priming participants with the concept of future generations increases how responsible they feel for reducing their contribution to climate change.

***Associations with Demographic Indicators***

When examining the correlations between the different types of responsibility and demographic indicators split by the order in which measures were shown to participants, we see that for both groups responsibility to future generations was correlated with political ideology less strongly than all other types of responsibility (see Table S14). Comparing these correlation coefficients with a Fisher’s Z test, suggested that in all cases this association was significantly weaker (*p*s ≤ .008) except for the comparison with responsibility to reduce carbon emissions for the group in which responsibility to future generations was shown first (*Z* = 1.66, *p* = .097). However, the one-tailed test is significant (*Z* = 1.66, *p* = .048), and this effect fall in the pre-registered hypothesized direction.

**Table S14**

*Bivariate Correlations Split by Order Group*

| RFG shown first | Future Generations | Global Warming | Carbon Emissions | Greenhouse Gas Emissions |
| --- | --- | --- | --- | --- |
| Ideology | -0.36*** | -0.52*** | -0.48*** | -0.52*** |
| Age | 0.03 | 0.00 | -0.01 | 0.01 |
| Religion | 0.00 | -0.14* | -0.14*** | -0.14* |
| Income | 0.11 | 0.00 | 0.05 | 0.01 |
| Education | 0.17 | 0.11 | 0.11 | 0.12 |
| RCC shown last | Future Generations | Global Warming | Carbon Emissions | Greenhouse Gas Emissions |
| Ideology | -0.43*** | -0.61*** | -0.61*** | -0.59*** |
| Age | -0.12* | -0.09 | -0.11 | -0.10 |
| Religion | -0.16** | -0.25*** | -0.27*** | -0.26*** |
| Income | 0.02 | -0.09 | -0.04 | -0.04 |
| Education | 0.08 | 0.10 | 0.14* | 0.15** |

**Note.** **p* < .05, ***p* < .01, *** *p* < .001.

***Associations with Other Responsibility Types***

All types of responsibility were strongly related to each other (see Table S15), suggesting that those who felt responsible to future generations also felt responsible to reduce global warming, carbon emissions, and greenhouse emissions.

**Table S15**

*Bivariate Correlations Between All Responsibility Types for Those Who Saw the Measure for Future Generations First (Below the Diagonal) or Last (Above the Diagonal)*

|  | Future Generations | Global Warming | Carbon Emissions | Greenhouse Emissions |
| --- | --- | --- | --- | --- |
| Future Generations | -- | 0.69 | 0.72 | 0.71 |
| Global Warming | 0.60 | -- | 0.91 | 0.89 |
| Carbon Emissions | 0.58 | 0.87 | -- | 0.93 |
| Greenhouse Emissions | 0.62 | 0.90 | 0.89 | -- |

**Note.** All *p*s < .001.

***RFG and Proenvironmental Outcomes*** Due to the observed order effects, and the strong correlation between responsibility to future generations and each other responsibility type, we ran analyses only for responsibility to future generations independently from other responsibility types. Those who felt responsible to protect future generations expressed significantly more engagement in environmental movement activism, personal conservation behaviors, support for conservation policies, environmental concern, and significantly less support for protecting the environment based on anthropocentric reasons and less support for population growth policies. All associations remained significant after adjusting for demographic covariates except for the association with support for population growth policies.

**Table S16**

*Linear Regression Models With and Without Covariates for RFG as the Predictor*

|  | RFG only | | | |  | RFG and covariates | | | |
| --- | --- | --- | --- | --- | --- | --- | --- | --- | --- |
| Outcome | β | Lower C.I. | Upper C.I. | Adj. R^2^ |  | β | Lower C.I. | Upper C.I. | Adj. R^2^ |
| Support for population growth policies | -0.15 | -0.31 | -0.09 | 0.02 |  | -.03 | -0.16 | 0.07 | 0.12 |
| Environmental concern | 0.45* | 0.30 | 0.42 | 0.20 |  | 0.35* | 0.22 | 0.35 | 0.26 |
| Anthropogenic concern | -0.35* | -0.35 | -0.23 | 0.12 |  | -0.24* | -0.26 | -0.13 | 0.27 |
| Personal conservation behaviors | 0.40* | 0.28 | 0.41 | 0.16 |  | 0.34* | 0.22 | 0.36 | 0.20 |
| Support for conservation policies | 0.58* | 0.54 | 0.69 | 0.34 |  | 0.42* | 0.37 | 0.51 | 0.50 |
| Environmental threat | 0.53* | 0.49 | 0.64 | 0.28 |  | 0.37* | 0.32 | 0.46 | 0.48 |

**Note.** **p* < .001. Adj. = Adjusted.

**Discussion**

Study S5 replicated part of the results of Study 3B. Specifically, results suggest that how responsible people feel for protecting future generations is less strongly related to a person’s political ideology when compared to how responsible they feel towards reducing global warming, greenhouse gas emissions, and carbon emissions. This evidence suggests that regardless of how responsibility towards reducing climate change is captured, this responsibility is more strongly related to a person’s political ideology, a major barrier to proenvironmental engagement (in the U.S.). Further, our results also suggest that responsibility to future generations robustly relates to different forms of proenvironmental engagement. Thus, even though we were not able to compare the overall endorsement of the different types of responsibility due to a significant order effect, we replicated and expanded upon the findings of Study 3A. Importantly, the significant order effect suggests that when people are asked to report their responsibility to future generations first, they then reported a higher level or responsibility to reduce global warming, carbon emissions and greenhouse emissions. Although unexpected and not pre-registered, this finding could suggest that making salient one’s responsibility to future generations could increase their general responsibility to reduce climate change (an intergenerational issue at its heart). However, to verify this claim, concrete experimental evidence is required.

**Study 3B – Supplementary Analyses**

**Table S17**

*Frequencies for those who differed in the two types of responsibility across all frames*

|  | Reducing Climate Change Higher | | Both Endorsed Equally | | Protecting future generations higher | |
| --- | --- | --- | --- | --- | --- | --- |
| Frame | N | % | N | % | N | % |
| Global Warming | 109 | 24.89 | 101 | 23.06 | 228 | 52.05 |
| Greenhouse Gas Emissions | 99 | 22.05 | 108 | 24.05 | 242 | 53.90 |
| Carbon Emissions | 84 | 18.88 | 76 | 17.08 | 285 | 64.04 |
| Climate Change | 95 | 21.21 | 86 | 19.2 | 267 | 59.59 |

**Table S18**

*Bivariate Correlations for All Measures for Republicans, Independents and Democrats*

| Republicans (N = 604) | 1 | 2 | 3 | 4 | 5 | 6 |
| --- | --- | --- | --- | --- | --- | --- |
| 1. Responsibility to Future Generations | -- |  |  |  |  |  |
| 2. Responsibility to Reduce Climate Change | 0.63 | -- |  |  |  |  |
| 3. Environmental Concern | 0.43 | 0.52 | -- |  |  |  |
| 4. Environmental Threat | 0.46 | 0.64 | 0.65 | -- |  |  |
| 5. Personal Conservation Behaviors | 0.53 | 0.55 | 0.54 | 0.50 | -- |  |
| 6. Environmental Movement Activism | 0.52 | 0.67 | 0.44 | 0.63 | 0.46 | -- |
| 7. Support for Interventionist Policies | 0.48 | 0.62 | 0.49 | 0.64 | 0.47 | 0.58 |
| Independents (N = 544) | 1 | 2 | 3 | 4 | 5 | 6 |
| 1. Responsibility to Future Generations | -- |  |  |  |  |  |
| 2. Responsibility to Reduce Climate Change | 0.74 | -- |  |  |  |  |
| 3. Environmental Concern | 0.47 | 0.52 | -- |  |  |  |
| 4. Environmental Threat | 0.51 | 0.65 | 0.61 | -- |  |  |
| 5. Personal Conservation Behaviors | 0.53 | 0.58 | 0.55 | 0.48 | -- |  |
| 6. Environmental Movement Activism | 0.54 | 0.67 | 0.45 | 0.58 | 0.46 | -- |
| 7. Support for Interventionist Policies | 0.46 | 0.57 | 0.50 | 0.67 | 0.39 | 0.51 |
| Democrats (N = 621) | 1 | 2 | 3 | 4 | 5 | 6 |
| 1. Responsibility to Future Generations | -- |  |  |  |  |  |
| 2. Responsibility to Reduce Climate Change | 0.74 | -- |  |  |  |  |
| 3. Environmental Concern | 0.42 | 0.46 | -- |  |  |  |
| 4. Environmental Threat | 0.35 | 0.42 | 0.67 | -- |  |  |
| 5. Personal Conservation Behaviors | 0.47 | 0.52 | 0.56 | 0.49 | -- |  |
| 6. Environmental Movement Activism | 0.46 | 0.50 | 0.38 | 0.33 | 0.45 | -- |
| 7. Support for Interventionist Policies | 0.28 | 0.33 | 0.48 | 0.51 | 0.39 | 0.31 |

**Note.** For all estimates, *p* < .001

**Table S19**

*Linear Regression Models With and Without Covariates For RCC (Collapsed Across Frames)*

|  | RCC only | | | |  | RCC and covariates | | | |
| --- | --- | --- | --- | --- | --- | --- | --- | --- | --- |
| Outcome | β | Lower C.I. | Upper C.I. | Adj. R^2^ |  | β | Lower C.I. | Upper C.I. | Adj. R^2^ |
| Support for interventionist conservation policies | 0.63* | .030 | .034 | .40 |  | 0.46* | .022 | .025 | .49 |
| Environmental concern | 0.57* | .018 | .021 | .33 |  | 0.49* | .015 | .018 | .35 |
| Environmental threat | 0.67* | .030 | .033 | .45 |  | 0.54* | .023 | .027 | .52 |
| Personal conservation behaviors | 0.58* | .022 | .025 | .33 |  | 0.36* | .021 | .025 | .36 |
| Environmental Movement Activism | 0.68* | .036 | .040 | .47 |  | 0.61* | .031 | .036 | .49 |

**Note.** Adj = Adjusted. **p* < .001.

**Forced Choice**

To examine whether a similar pattern of results would emerge in a forced-choice format, we asked participants: “From the following two choices, if you could only pick one, which one do you feel more responsible for?”. They were given the following choices, either (a) protecting future generations or (b) reducing global warming/greenhouse gas emissions/carbon emissions/global warming. For each of the four frames, participants chose to protect future generations more frequently than to reduce climate change (58% compared to 42% for the comparison with climate change, 72% compared to 28% for the comparison with carbon emissions, 67% compared to 33% compared to greenhouse gas emissions, and 57% compared to 43% for global warming).

When we collapsed across all frames, a chi-square test suggested that there was a significant difference in how likely participants from different political backgrounds tended to choose the “protect future generations option”. Importantly, Democrats were equally likely to choose either option, while Independents and Republicans were more likely to select the “protect future generations option”, *χ^2^* (2) = 103, 26, *p* < .001, V = .24. Phi = .24.

**Figure S5**

*Bar Graph Depicting the Percentage of Each Choice (Collapsed Across Frames) for each Group*

**Study 4 – Supplementary Information**

**Table S20**

*Bivariate Correlations Between Demographic Indicators and All Outcome Measures. Coefficients Above* r = .10 *Are Significant at p < .001.*

|  | Age | Income level | Political Ideology | Education level | Religiosity |
| --- | --- | --- | --- | --- | --- |
| Responsibility Future Generations | **0.11** | 0.09 | **-0.19** | 0.09 | 0.06 |
| Responsibility for reducing climate change | 0.02 | 0.07 | **-0.34** | 0.09 | -0.04 |
| Donate to Charity | **0.15** | 0.07 | -0.05 | 0.05 | 0.07 |
| Intentions | 0.03 | 0.07 | **-0.21** | **0.12** | 0.05 |
| Policy Support | **-0.14** | 0.01 | **-0.58** | 0.05 | **-0.30** |

**Table S21**

*Results With Demographic Covariates Accounted For*

| **Policy Support** | **b** | **β** | **SE** | **t** | **p** | **95% C.I.** | |
| --- | --- | --- | --- | --- | --- | --- | --- |
| Intercept | 5.84 | 0.00 | .06 | 96.97 | <.001 | 5.72 | 5.95 |
| Legacy | 0.09 | 0.03 | 0.05 | 1.69 | .092 | -0.01 | 0.19 |
| Letter | 0.14 | 0.05 | 0.05 | 2.71 | .007 | 0.04 | 0.24 |
| Longtermism | 0.09 | 0.03 | 0.05 | 1.83 | .068 | -0.01 | 0.19 |
| Sacrifice | -0.01 | 0.00 | 0.05 | -0.20 | .838 | -0.11 | 0.09 |
| Video | 0.11 | 0.04 | 0.05 | 2.18 | .030 | 0.01 | 0.21 |
| Ideology | -0.35 | -0.56 | 0.01 | -36.29 | <.001 | -0.37 | -0.34 |
| Age | 0.00 | -0.02 | 0.00 | -1.33 | .184 | 0.00 | 0.00 |
| Religiosity | -0.08 | -0.09 | 0.01 | -5.68 | <.001 | -0.11 | -0.05 |
| **Intentions** | **b** | **β** | **SE** | **t** | **p** | **95% C.I.** | |
| Intercept | 2.87 | 0.00 | 0.08 | 37.53 | <.001 | 2.72 | 3.02 |
| Legacy | 0.17 | 0.06 | 0.06 | 2.86 | .004 | 0.05 | 0.29 |
| Letter | 0.53 | 0.18 | 0.06 | 8.52 | <.001 | 0.41 | 0.65 |
| Longtermism | 0.22 | 0.08 | 0.06 | 3.75 | <.001 | 0.11 | 0.34 |
| Sacrifice | 0.21 | 0.08 | 0.06 | 3.53 | <.001 | 0.09 | 0.33 |
| Video | 0.20 | 0.07 | 0.06 | 3.33 | .001 | 0.08 | 0.31 |
| Ideology | -0.14 | -0.22 | 0.01 | -12.71 | <.001 | -0.16 | -0.11 |
| Education | 0.09 | 0.11 | 0.01 | 6.38 | <.001 | 0.06 | 0.11 |
| **Donations** | **b** | **β** | **SE** | **t** | **p** | **95% C.I.** | |
| Intercept | 2.34 | 0.00 | 0.22 | 10.87 | <.001 | 1.92 | 2.76 |
| Legacy | 0.27 | 0.03 | 0.19 | 1.43 | .154 | -0.10 | 0.64 |
| Letter | 0.48 | 0.05 | 0.19 | 2.49 | .013 | 0.10 | 0.86 |
| Longtermism | 0.16 | 0.02 | 0.19 | 0.86 | .391 | -0.20 | 0.52 |
| Sacrifice | 0.14 | 0.02 | 0.19 | 0.73 | .468 | -0.23 | 0.51 |
| Video | 0.26 | 0.03 | 0.19 | 1.39 | .165 | -0.11 | 0.62 |
| Ideology | -0.15 | -0.08 | 0.03 | -4.30 | <.001 | -0.21 | -0.08 |
| Age | 0.04 | 0.17 | 0.00 | 9.25 | <.001 | 0.03 | 0.04 |
| **RCC** | **b** | **β** | **SE** | **t** | **p** | **95% C.I.** | |
| Intercept | 5.97 | 0.00 | 0.07 | 81.66 | <.001 | 5.82 | 6.11 |
| Legacy | 0.18 | 0.05 | 0.08 | 2.15 | .0315 | 0.02 | 0.34 |
| Letter | 0.39 | 0.10 | 0.08 | 4.60 | <.001 | 0.22 | 0.55 |
| Longtermism | 0.20 | 0.05 | 0.08 | 2.54 | .011 | 0.05 | 0.36 |
| Sacrifice | 0.11 | 0.03 | 0.08 | 1.33 | .1841 | -0.05 | 0.27 |
| Video | 0.19 | 0.05 | 0.08 | 2.38 | .0173 | 0.03 | 0.35 |
| Ideology | -0.30 | -0.35 | 0.01 | -20.95 | <.001 | -0.33 | -0.27 |
| **RFG** | **b** | **β** | **SE** | **t** | **p** | **95% C.I.** | |
| Intercept | 5.20 | 0.00 | 0.09 | 61.09 | <.001 | 5.04 | 5.37 |
| Legacy | 0.27 | 0.08 | 0.07 | 3.62 | <.001 | 0.12 | 0.41 |
| Letter | 0.35 | 0.10 | 0.08 | 4.63 | <.001 | 0.20 | 0.50 |
| Longtermism | 0.17 | 0.05 | 0.07 | 2.31 | .021 | 0.03 | 0.31 |
| Sacrifice | 0.27 | 0.08 | 0.07 | 3.57 | <.001 | 0.12 | 0.41 |
| Video | 0.20 | 0.06 | 0.07 | 2.77 | .006 | 0.06 | 0.35 |
| Ideology | -0.18 | -0.24 | 0.01 | -13.54 | <.001 | -0.21 | -0.16 |
| Age | 0.01 | 0.15 | 0.00 | 8.80 | <.001 | 0.01 | 0.02 |

**Comparison of Responsibility Types**

We also compared the two types of responsibility (for climate change and for future generations), for the control condition (where no manipulation of these measures was observed) and for each political party (in the control condition). The results summarized below replicated the findings from our previous studies. Overall, participants in the control condition reported feeling more responsible to future generations than for reducing climate change, *t*(551) = 3.91, *p* < .001, *d* = .11, ΔM = .15 95% C.I. [0.08, 0.24]. A similar pattern of results was observed for Republicans, *t*(82) = 3.95, *p* < .001, *d* = .34, ΔM = 0.57 95% C.I. [0.29, 0.86], Independents, *t*(156) = 2.57, *p* = .011, *d* = .15, ΔM = 0.20 95% C.I. [0.05, 0.35], but not for Democrats, *t*(290) = 0.475, *p* = .639.

**Correlation Between Outcomes**

As expected, responsibility to future generations was positively related to all other outcomes.

**Table S22**

*Bivariate Correlations Between The Outcome Measures*

|  | 1 | 2 | 3 | 4 | 5 |
| --- | --- | --- | --- | --- | --- |
| 1. RFG | -- |  |  |  |  |
| 2. RCC | 0.75 | -- |  |  |  |
| 3. Donations to Charity | 0.28 | 0.26 | -- |  |  |
| 4. Proenvironmental Intentions | 0.53 | 0.58 | 0.24 | -- |  |
| 5. Proenvironmental Policy Support | 0.41 | 0.61 | 0.15 | 0.36 | -- |

**Note.** All coefficients are significant at *p* < .001.

**Figure S6**

*Pre-registered Path Model with Responsibility to Future Generations and for Reducing Climate Change as Serial Mediators*

**
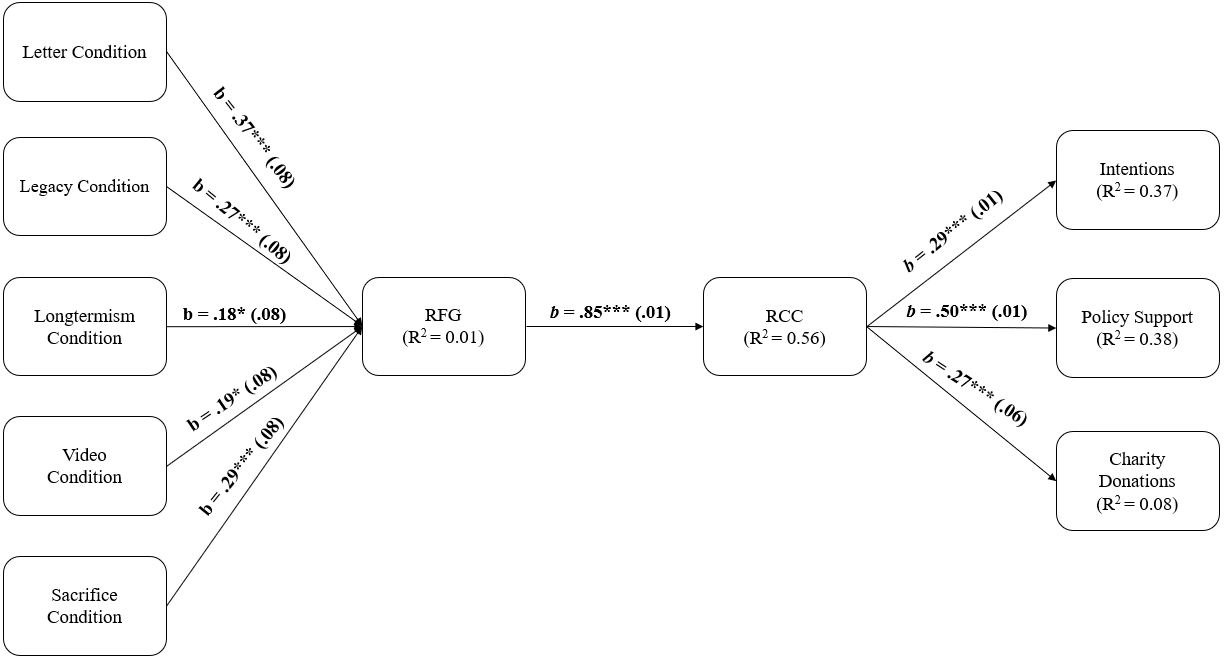
**

**Table S23**

*Indirect Effects Tested in Figure S6*

| Indirect effect | *b* | *SE* | *p* |
| --- | --- | --- | --- |
| RFG 🡪 RCC 🡪Donations | 0.23 | 0.05 | <.001 |
| RFG 🡪RCC 🡪Intentions | 0.24 | 0.01 | <.001 |
| RFG 🡪RCC 🡪Policy Support | 0.43 | 0.01 | <.001 |
| Video 🡪RFG 🡪RCC | 0.16 | 0.06 | 0.012 |
| Sacrifice 🡪RFG 🡪RCC | 0.24 | 0.06 | <.001 |
| Longtermism 🡪RFG 🡪RCC | 0.15 | 0.06 | 0.017 |
| Letter 🡪RFG 🡪RCC | 0.31 | 0.07 | <.001 |
| Legacy 🡪RFG 🡪RCC | 0.23 | 0.06 | <.001 |
| Video 🡪RFG 🡪RCC 🡪Donations | 0.14 | 0.05 | 0.013 |
| Video 🡪RFG 🡪RCC --> Intentions | 0.09 | 0.04 | 0.015 |
| Video 🡪RFG 🡪RCC 🡪Policy Support | 0.08 | 0.04 | 0.047 |
| Sacrifice 🡪RFG 🡪RCC 🡪Donations | 0.17 | 0.06 | 0.003 |
| Sacrifice 🡪RFG 🡪RCC 🡪Intentions | 0.09 | 0.04 | 0.013 |
| Sacrifice 🡪RFG 🡪RCC 🡪Policy Support | 0.04 | 0.04 | 0.266 |
| Longtermism 🡪RFG -> RCC 🡪Donations | 0.15 | 0.05 | 0.007 |
| Longtermism 🡪RFG -> RCC 🡪Intentions | 0.10 | 0.04 | 0.004 |
| Longtermism 🡪RFG -> RCC 🡪Policy Support | 0.11 | 0.04 | 0.005 |
| Letter 🡪RFG 🡪RCC 🡪Donations | 0.28 | 0.06 | <.001 |
| Letter 🡪RFG 🡪RCC 🡪Intentions | 0.19 | 0.04 | <.001 |
| Letter 🡪RFG 🡪RCC 🡪Policy Support | 0.18 | 0.04 | <.001 |
| Legacy 🡪RFG 🡪RCC 🡪Donations | 0.17 | 0.05 | 0.002 |
| Legacy 🡪RFG 🡪RCC 🡪Intentions | 0.1 | 0.04 | 0.005 |
| Legacy 🡪RFG 🡪RCC 🡪Policy Support | 0.07 | 0.04 | 0.074 |

**Note.** RFG = Responsibility to Future Generations, RCC = Responsibility to Reduce Climate Change.

**Analyses Requested by Reviewers**

**Table S24**

*Exploratory Factor Analysis for RFG and RCC Items in Study 3A*

| Item | RCC | RFG |
| --- | --- | --- |
| When we make decisions today we should take into consideration how our actions contribute to climate change | 0.95 |  |
| People living today have an obligation to reduce climate change | 0.93 |  |
| When deciding how to live, I have a duty to consider the impact of my actions on climate change | 0.90 |  |
| I feel a personal responsibility to do my best to reduce climate change | 0.84 |  |
| I’m willing to sacrifice aspects of my own life if it will help reduce the negative impacts of climate change | 0.80 |  |
| I feel a personal responsibility to do my best to protect future generations |  | 0.95 |
| When deciding how to live, I have a duty to consider the impact of my actions on future generations |  | 0.89 |
| I’m willing to sacrifice aspects of my own life if it will help people living in the future |  | 0.84 |
| When we make decisions today we should take into consideration how our actions influence future generations |  | 0.75 |
| People living today have an obligation to protect future generations |  | 0.74 |
| Variance Explained | 90% | 10% |

**Note.** Loadings below .20 are not shown.

**Table S25**

*Moderation of the association between RFG and each proenvironmental outcome by age and political ideology estimated with the PROCESS Macro, Model 1, with 10,000 bootstraps*

| Outcome | Moderator (W) | Interaction | Effect of RFG | Effect of W | Effect at low levels of W | Effect at high levels of W | R^2^ |
| --- | --- | --- | --- | --- | --- | --- | --- |
| EC | Age | <.001 (<.001) | .02*** (.001) | -.002 (.01) | -- | -- | 0.23 |
| ET | Age | .001*** (<.001) | .01*** (.004) | -.03***(.01) | .02*** (.001) | .03*** (.002) | 0.25 |
| PCB | Age | <.001 (<.001) | .03*** (.003) | .02*** (.005) | -- | -- | 0.31 |
| EMA | Age | <.001 (.001) | .03*** (.004) | -.02*** (.01) | -- | -- | 0.31 |
| CP | Age | .001*** (<.001) | .01** (.01) | -.04*** (.01) | .02*** (.002) | .03*** (.002) | 0.24 |
| EC | Conservatism | <.001 (.001) | .02*** (.002) | -.19*** (.03) | -- | -- | 0.31 |
| ET | Conservatism | .003*** (.001) | .01* (.003) | -.58*** (.04) | .01*** (.001) | .03*** (.001) | 0.44 |
| PCB | Conservatism | .001* (.001) | .02*** (.003) | -.16*** (.04) | .02*** (.002) | .03*** (.001) | 0.31 |
| EMA | Conservatism | <.001 (.001) | .03*** (.003) | -.27*** (.05) | -- | -- | 0.39 |
| CP | Conservatism | .003*** (.001) | .01** (.003) | -.62*** (.05) | .01*** (.002) | .03*** (.002) | 0.45 |

**Note.** **p* < .05, ** *p* <.01, *** *p* < .001.
EC = Environmental Concern; ET = Environmental Threat; PCB = Personal Conservation Behaviors; EMA = Environmental Movement Activism; CP = Conservation Policies

**Table S26**

*Omnibus Tests of the Exploratory Analyses Comparing the Treatment Conditions*

| Outcome | Omnibus Test |
| --- | --- |
| RFG | *F*(4, 2618) = 2.01, *p* = .091 |
| RCC | *F*(4, 2618) = 2.87, *p* = .022, η^2^_p_ = .004 |
| Policy Support | *F*(4, 2618) = 1.65, *p* = .158 |
| Intentions | *F*(4, 2618) = 10.43, *p* < .001, η^2^_p_ = .016 |
| Donations | *F*(4, 2618) = 0.97, *p* = .423 |

**Table S27**

*Post-hoc comparisons of the Exploratory Analyses Comparing the Treatment Conditions*

| Outcome | Comparison | b | SE | t | p |
| --- | --- | --- | --- | --- | --- |
| **Intentions** | **Letter vs Legacy** | **0.36** | **0.06** | **5.55** | **<.001** |
| **Intentions** | **Letter vs Longtermism** | **0.30** | **0.06** | **4.69** | **<.001** |
| **Intentions** | **Letter vs Sacrifice** | **0.32** | **0.07** | **4.86** | **<.001** |
| **Intentions** | **Letter vs Video** | **0.35** | **0.06** | **5.42** | **<.001** |
| Intentions | Legacy vs Longtermism | -0.06 | 0.06 | -0.95 | .341 |
| Intentions | Legacy vs Sacrifice | -0.04 | 0.06 | -0.67 | .504 |
| Intentions | Legacy vs Video | -0.01 | 0.06 | -0.19 | .848 |
| Intentions | Longtermism vs Sacrifice | 0.02 | 0.06 | 0.27 | .788 |
| Intentions | Longtermism vs Video | 0.05 | 0.06 | 0.77 | .442 |
| Intentions | Sacrifice vs Video | 0.03 | 0.06 | 0.49 | .627 |
| **RCC** | **Letter vs Legacy** | **0.23** | **0.09** | **2.57** | **.010** |
| RCC | Letter vs Longtermism | 0.17 | 0.09 | 1.95 | .052 |
| **RCC** | **Letter vs Sacrifice** | **0.28** | **0.09** | **3.08** | **.002** |
| **RCC** | **Letter vs Video** | **0.23** | **0.09** | **2.60** | **.009** |
| RCC | Legacy vs Longtermism | -0.06 | 0.09 | -0.67 | .504 |
| RCC | Legacy vs Sacrifice | 0.05 | 0.09 | 0.55 | .583 |
| RCC | Legacy vs Video | 0.00 | 0.09 | 0.01 | .994 |
| RCC | Longtermism vs Sacrifice | 0.11 | 0.09 | 1.22 | .222 |
| RCC | Longtermism vs Video | 0.06 | 0.09 | 0.69 | .493 |
| RCC | Sacrifice vs Video | -0.05 | 0.09 | -0.55 | .584 |

**Note.** Bolded effects denote significant differences.

**References**

Milfont, T., & Duckitt, J. (2010). The environmental attitudes inventory: A valid and reliable measure to assess the structure of environmental attitudes. *Journal of Environmental Psychology, 30*(1). 80-94. <https://doi.org/10.1016/j.jenvp.2009.09.001>

Sparkman, G., Geiger, N. & Weber, E.U. (2022). Americans experience a false social reality by underestimating popular climate policy support by nearly half. *Nature Communications, 13*, 4779. <https://doi.org/10.1038/s41467-022-32412-y>

Syropoulos, S., & Markowitz, E. M. (2022). Perceived responsibility to address climate change consistently relates to increased pro-environmental attitudes, behaviors and policy support: Evidence across 23 countries. *Journal of Environmental Psychology, 83*, 101868. <https://doi.org/10.1016/j.jenvp.2022.101868>

Syropoulos, S., & Markowitz, E. M. (2021). Perceived responsibility towards future generations and environmental concern: Convergent evidence across multiple outcomes in a large, nationally representative sample. *Journal of Environmental Psychology, 76*, 101651. <https://doi.org/10.1016/j.jenvp.2021.101651>

Syropoulos, Watkins, H. M., Shariff, A. F., Hodges, S. D. S., & Markowitz, E. M. (2020). The role of gratitude in motivating intergenerational environmental stewardship. *Journal of Environmental Psychology, 72*, 101517. <https://doi.org/10.1016/j.jenvp.2020.101517>
